# Designing for all: A scoping review and critical analysis on how digital mental health interventions serve (or fail) diverse women

**DOI:** 10.1016/j.invent.2026.100981

**Published:** 2026-07-16

**Authors:** Ruixuan Zhang, Mark de Reuver, Angela D.R. Smith, Nic Orchard, Caroline Figueroa

**Affiliations:** a Faculty of Technology, Policy and Management, Delft University of Technology, Jaffalaan 5, 2628BX, Delft, the Netherlands; b Department of Black Studies, University of Rochester

**Keywords:** Digital mental health, Human-centered design, Diversity and inclusivity, Health equity, Intersectionality

## Abstract

**Background:**

Women face unique and diverse mental health challenges and therefore require tailored interventions that address their specific needs and lived realities. Research has shown a lack of digital interventions designed for and with women from marginalized backgrounds, such as minority racial/ethnic backgrounds or with low socioeconomic status. If Digital Mental Health Interventions (DMHI) are not designed with active involvement of women from diverse backgrounds, they may fail to reach their public health objectives, which potentially exacerbates existing health disparities. How Human-Centered design methods are applied and reported, and whether women from diverse backgrounds are involved in the design and development of Digital Mental Health Interventions (DMHI), is under-researched.

**Objective:**

This study examined the methods and frameworks used in the design and development processes of DMHIs, whether and how studies include women from diverse backgrounds, and whether they tailor to their diverse needs related to intersectional identities (e.g., race/ethnicity, socioeconomic status, age) in the design and development process.

**Methods:**

We conducted a scoping review following the PRISMA-ScR guidelines. The databases included are Scopus, PubMed and IEEE Xplore, and the databases are searched from the inception until the 31st of May 2026. We included 77 articles that described the design process of digital mental health solutions for women.

**Results:**

Among the 77 reviewed studies, 18 (23.4%) did not explicitly state their design methods, and most of them provided limited information on the characteristics of the included populations. Only 16 (20.8%) studies consistently involved users across all design stages. Intersectional identities were considered in only 26 (33.8%) studies. We observed an overrepresentation of research in higher-income countries, an underrepresentation of women from racial/ethnic minority backgrounds, a narrow age range of participants, and a lack of consideration of intersectional identities.

**Conclusions:**

Our findings reveal critical gaps in the development of DMHIs for women, including the superficial reporting and application of Human-Centered Design methods in the design process, limited user involvement, and a lack of consideration of diversity, inclusivity and intersectionality. Future research should emphasize active involvement of end-users from the earliest design phases onwards and adopting an intersectional lens in their design processes. We propose a research agenda for better reporting and applying HCD methods in future DMHI research, towards designing diverse, inclusive and equitable digital solutions for all women.

**Registry and registry number for systematic reviews or meta-analyses:**

A protocol of this study is pre-registered at Open Science Framework (DOI 10.17605/OSF.IO/WC79P).

## Highlights

• Reporting and application of Human-Centered Design methods remain inconsistent in women's DMHI development.
• Many studies skip early user involvement and engage users only in prototype evaluation or RCTs.
• Women from marginalized backgrounds remain underrepresented in studies.
• Intersectionality is rarely considered in DMHI design, limiting cultural relevance and equity.
• A research agenda is proposed for better HCD reporting and use, and more inclusive DMHI design.

## 1 Introduction

One in five individuals globally suffers from mental health disorders, with women twice as likely to experience a mental health disorder like depression or anxiety as men (Steel et al., 2014; Kuehner, 2016). Women, particularly those with minoritized cultural identities, face unique and intersecting challenges in maintaining mental well-being (Liu et al., 2023). For example, minoritized race/ethnicity, low socioeconomic status, immigration backgrounds, and limited digital health literacy heighten the risks of experiencing mental health disorders (Johnson et al., 2019).

At the same time, women's cultural identities shape their experiences and needs in mental well-being support. For example, research highlights the significant role of religion and spirituality (R/S) in supporting the mental well-being of Muslim women (Douki et al., 2007; Hamdan, 2009; Saherwala et al., 2021). Religion and faith often positively influence Muslim women's self-esteem, emotional health, life satisfaction, and a sense of meaning and purpose (Hamdan, 2009). Moreover, community support based on R/S is a critical component in strengthening mental and emotional health among many Muslim women (Mohr et al., 2019). Therefore, culturally relevant care that respects religious beliefs and acknowledges the importance of family and community is essential for supporting the mental well-being of Muslim women.

Another example is that Indigenous women frequently experience historical trauma stemming from colonization, and they often deeply connect with their land and nature and have a strong sense of connection to ancestral history and community (Wilson, 2005; Paradies, 2016). Consequently, Indigenous women often find community-based and ceremony-related support more effective compared to individualized therapy (Varcoe et al., 2019). These examples stress the urgent need to provide culturally sensitive mental health support that addresses the diverse needs of women.

Meanwhile, digital mental health interventions (DMHIs) deliver mental health supports through technologies, such as mobile apps, websites, wearable devices, VR/AR, and video therapy (Löchner et al., 2025). Recent research has shown that DMHIs are promising tools for improving health outcomes, increasing access, and improving cost-efficiency, especially for those who face geographic barriers (Becker and Torous, 2019; Lehtimaki et al., 2021; Yeo et al., 2023). However, their design and implementation often fail to adequately serve marginalized populations, who remain underrepresented in digital mental health research and interventions (Ellis et al., 2022; Bear et al., 2024).

The design process of Digital Mental Health Interventions (DMHIs) plays a significant role in their acceptability, usability, and user engagement. Research has shown that Human-Centered Design (HCD) approaches can promote inclusivity in health research if properly deployed (Holeman and Kane, 2019; Sherman et al., 2024). By integrating iterative collaboration with end users throughout the design process, HCD emphasizes that digital interventions align with individuals' needs, preferences, and real-world contexts (International Organization for Standardization (ISO), 2019). Additionally, HCD falls in a broader stream of methods that address inclusivity, such as user-centered design (UCD), participatory design (PD), and co-design. According to the International Standards of Human-centered Design for Interactive Systems (ISO, 2019), an ideal HCD approach should follow six key principles: (1) the design is grounded in a thorough understanding of users, their tasks, and their environments; (2) users are directly involved throughout every phase of design and development; (3) user-centered evaluation drives and refines the design; (4) the design process is iterative; (5) the design comprehensively addresses the user experience; and (6) multidisciplinary design teams contribute diverse skills and perspectives. HCD methods in digital health development have been shown to effectively improve user engagement and adherence, but only when consistently and correctly adopted in line with these principles (Johnston et al., 2022; Duffy et al., 2023).

Despite the growing recognition of HCD in the digital mental health domain, significant gaps remain in understanding how these methods are applied in practice and whether they truly adhere to their key principles. Originating in industrial design, HCD has been widely used by design agencies for decades, but is often inconsistently or misused in digital health. The rise of HCD in the digital health field was often driven by oversimplified assumptions about user preferences and behaviors, leading to digital health tools that failed to align with user needs (Holeman and Kane, 2019). Critics have argued that HCD is often used as a buzzword in digital health rather than a rigorously applied methodology, limiting its ability to ensure user-centered design practices that genuinely reflect participants' needs and promote health equity (Holeman and Kane, 2019). For example, whether HCD methods are consistently and effectively integrated into the development of DMHIs remains poorly studied.

Moreover, to extend the effects of HCD in digital mental health, diversity, inclusivity, and intersectionality must be actively incorporated, as these factors shape user needs and experiences. However, these considerations are not explicit components described in the HCD process (Almeida et al., 2020), and an intersectional lens is often lacking. The concept of intersectionality, first introduced by Kimberlé Williams Crenshaw in 1989, emerged from Black feminist scholarship as a critique of the “single-axis framework” in antidiscrimination law, which overlooked the compounded discrimination Black women faced due to both racism and sexism (Kimberlé, 1989). In recent years, it has become well-recognized that the interconnectedness of multiple identities, such as race/ethnicity, gender, socioeconomic status, and sexual orientation, shapes mental health and well-being (Rotz et al., 2021). For example, Rosenthal and Lobel (2018) found that Black and Latina women in the U.S. often experience overlapping discrimination based on race, class, and gender, which significantly worsens their mental health outcomes. Similarly, digital health access and needs depend not only on gender but also on factors such as socioeconomic status, race or ethnicity, and geographic location (Figueroa et al., 2021a). Thus, even when HCD is properly used, DMHI research may still fail to address the mental health needs of all women.

Given the urgent need to address HCD in DMHIs' development and to consider diversity and inclusivity, this study examines the current landscape of DMHIs for women. We identify critical gaps and propose a research agenda to enhance diversity and inclusivity, ultimately promoting equity in future digital mental health research. Specifically, this research examines how HCD methods are applied in DMHI development, the level of end-user involvement in designing interventions for women, and how diversity, inclusivity, and intersectionality are incorporated into the design process through an intersectionality lens. To address these concerns, we investigate the following research questions:

(1) Do studies use human-centered design methods, and how do they implement them?
(2) How do studies involve end users in their design phases?
(3) Do studies include racial/ethnic minoritized women, women with low socioeconomic status, or women with immigration backgrounds?
(4) Do studies consider the intersecting cultural identities of women in their design process?

Moreover, the hypotheses of this study are as follows:

(1) Most studies lack consistent adoption of Human-Centered Design methods that align with the design principles of these methods.
(2) Most studies lack end users' (women's) involvement in the design process of DMHIs or involve them only partially, i.e., only in randomized controlled trials or usability tests.
(3) Most studies lack significant representation of racial/ethnic minoritized participants and participants with low socioeconomic status in the design process of DMHIs.
(4) Most studies lack consideration of the intersecting cultural identities of women in their design process.

## 2 Methods

### 2.1 Study design

**Fig. 1:**
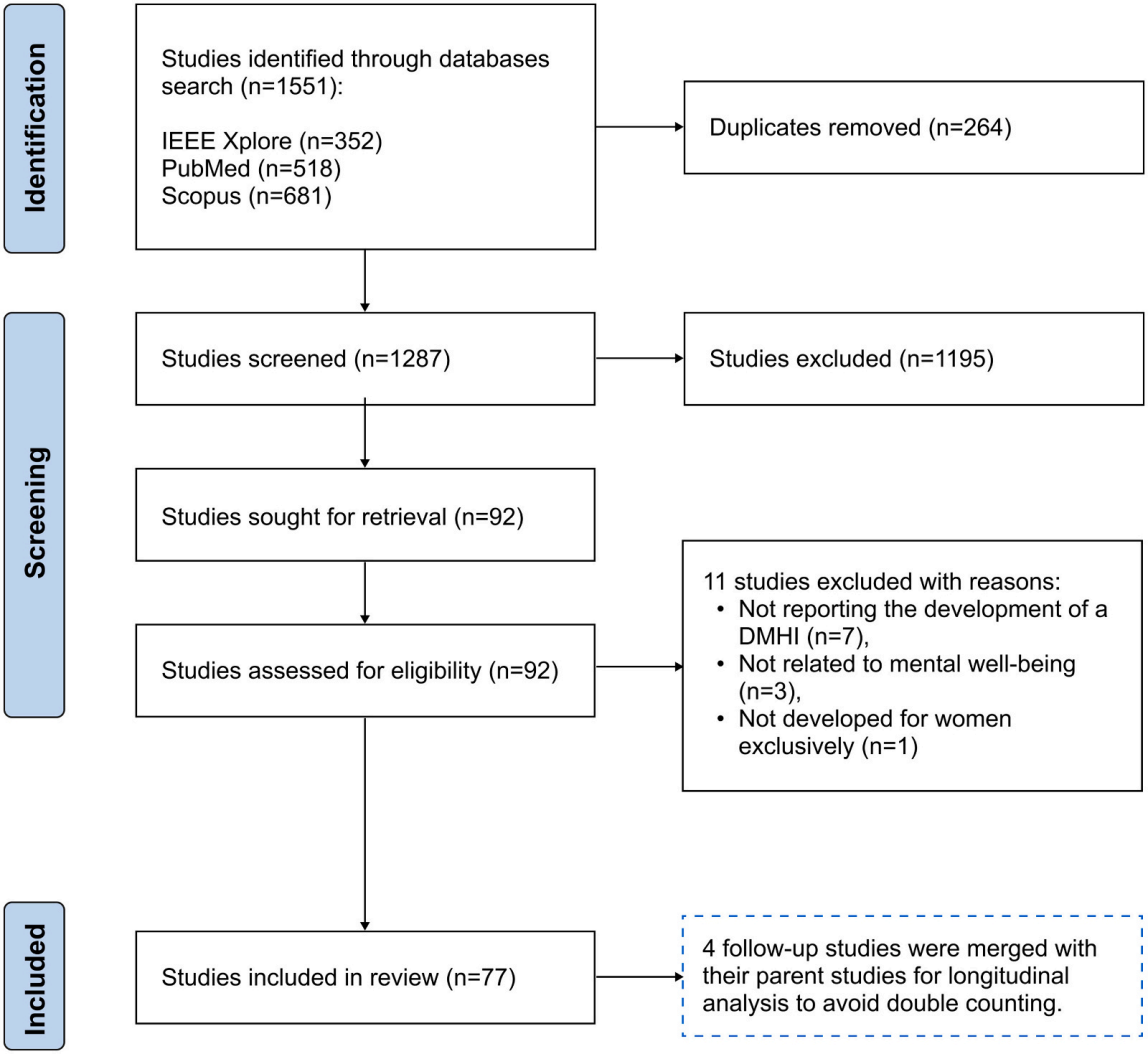
Flow chart of study procedure following the PRISMA-ScR guidelines.

**Fig. 2:**
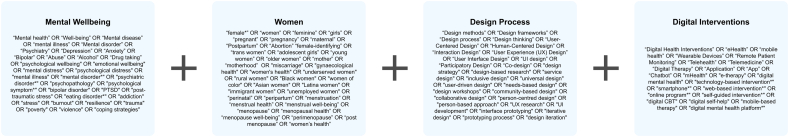
Query terms used in search.

To answer the four research questions, we conducted a scoping review because it is well-suited for exploring broad research topics and systematically mapping existing evidence to identify key concepts, gaps, and research priorities (Arksey and O'Malley, 2005). A protocol for this study was pre-registered on the Open Science Framework (DOI 10.17605/OSF.IO/WC79P). This review followed the steps outlined in the Preferred Reporting Items for Systematic Reviews and Meta-Analyses extension for Scoping Reviews (PRISMA-ScR) (Tricco et al., 2018). A completed PRISMA-ScR checklist is available in Appendix A. Fig. 1, Fig. 2 show the study process and the query terms, respectively. The full search terms used are also provided in Appendix B, Supplementary Table 1. The search included one multidisciplinary database (Scopus) and two discipline-specific databases (PubMed and IEEE Xplore). The initial search was conducted from March 2023 to January 2024 and was updated in May 2026 following peer review. The databases were searched for publications from 2014 to 2026. The screening and data extraction processes were carried out independently by 2–3 researchers, with any conflicts resolved through group discussions. The full list of references for all included studies, including related longitudinal study publications, can be found in Appendix B, Supplementary Table 2.

The inclusion criteria of this study are as follows:

1) Studies should be exclusively focused on women.
2) Studies should discuss mental well-being, mental health concerns, or disorders, including all articles related to mental health/mental well-being interventions. This includes but is not limited to preventive interventions, self-management/monitoring and self-care tools, clinical Interventions, behavioral well-being, support and counseling, social well-being, etc.
3) Studies should involve digital interventions (not limited to specific types).

The exclusion criteria are as follows:

1) Studies that are not exclusively focused on women
2) Studies that do not discuss mental well-being-related topics
3) Studies that do not involve digital interventions

### 2.2 Data extraction and synthesis

Table 1 lists the extracted data items and their descriptions. To structure the analysis, we adopted the Integrate, Design, Assess, and Share (IDEAS) framework by Mummah et al. (2016), which was developed to guide the development of digital behavioral health interventions. This framework innovatively combines the iterative, user-centered focus of design thinking with the evidence-based rigor of behavioral theory, making it particularly suited to developing mental health interventions. The IDEAS framework emphasizes active user involvement across all design and development stages of digital health interventions.

**Table 1:** Data items extracted from eligible studies.

Category	Data items
Study identification	Published year and country.
	Type of mental well-being problem targeted.
Design method	Adopted design methods that structure or guide the overall intervention design process.
	User involvement during the IDEATE stage, the type of activities, and the demographics of involved users.
	User involvement during the DESIGN stage, the type of activities, and the demographics of involved users.
	User involvement during the DESIGN stage, the type of activities, and the demographics of involved users.
Diversity, inclusivity, and intersectionality consideration	Consideration of intersecting identities.
	Does the intervention aim to represent the needs of marginalized populations (people with low SES, migration backgrounds, or minority race/ethnicity)?
	Does the study assess differences in outcomes by SES, immigration background, race, or ethnicity?
	Overview of demographics information of involved users, including age, race/ethnicity, immigration status, education levels, and annual income (or of their parents).

The IDEAS framework comprises four phases: Integrate, Design, Assess, and Share. During an initial round of data extraction, we observed that the included studies rarely reported activities related to the Share phase, such as dissemination, implementation, or long-term scaling of interventions. Consequently, this review focused primarily on the first three phases (Integrate, Design, and Assess), with particular attention to user involvement and diversity considerations throughout the design and development process. While this represents a partial application of the IDEAS framework, we considered this approach appropriate given the limited reporting of Share-related activities in the reviewed literature. However, this exclusion may limit insights into how DMHIs are disseminated, implemented, or sustained beyond the development and assessment stages.

The IDEAS framework comprises four phases: Integrate, Design, Assess, and Share.

Additionally, we investigated the demographic composition of participants, whether and how interventions aimed to address the needs of marginalized populations, and whether they assessed differences in outcomes based on SES, immigration background, race, or ethnicity. Finally, we examined whether studies mentioned tailoring to the needs of women according to intersectional identities. For example, whether they tailored DMHIs to meet the needs of females, while also considering needs related to belonging to a racial/ethnic minority group.

## 3 Results

### 3.1 Published year and geographic location distribution

**Fig. 3:**
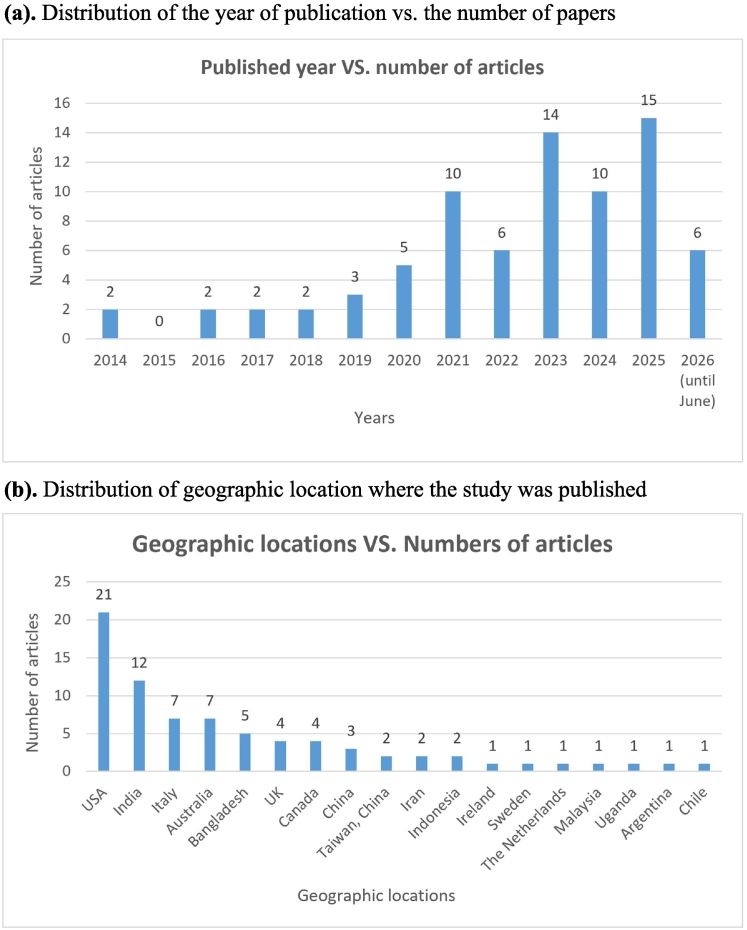
(a). Distribution of the year of publication vs. the number of papers. (b). Distribution of geographic location where the study was published.

This review includes eligible studies published from January 2013 to June 2026. Fig. 3(a) and (b) illustrates the distribution of studies by year of publication and geographic location, respectively. An increasing number of DMHI studies focused on women have been published over the past five years, particularly following the COVID-19 pandemic.

The included studies were conducted across 18 countries (Fig. 3(b)). Most studies originated from the United States (*n* = 21), followed by India (*n* = 12), Italy (*n* = 7), and Australia (n = 7). Based on the International Monetary Fund (IMF) classification of advanced, emerging, and developing economies (World Economic Outlook Database - Groups and Aggregates Information, 2025), the majority of studies (49/77, 62.0%) were conducted in advanced economies, while 30 studies (38.0%) came from emerging markets and developing economies, such as India, Iran, Uganda, and Bangladesh.

### 3.2 Type of mental well-being problem targeted

**Fig. 4:**
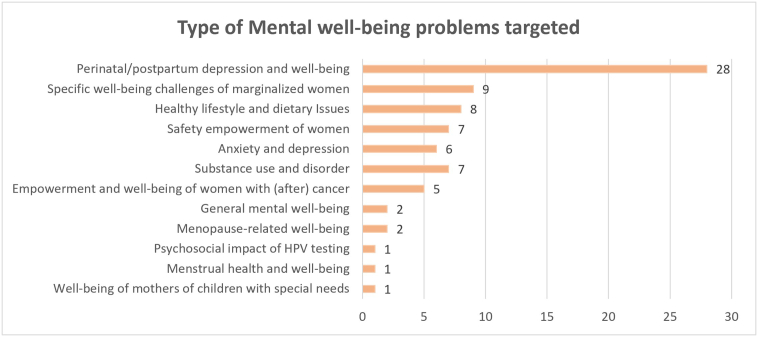
Types of mental well-being problems targeted.

Based on the full-text review, we classified nine categories of mental well-being problems targeted by the included articles (see Fig. 4). Of these categories, “Perinatal/postpartum depression and well-being” was the most common, accounting for 36.4% of the studies. Several sub-categories emerged within this category, such as the prevention of perinatal/postpartum depression, anxiety, and stress related to premature birth, post-abortion care, and subclinical depression symptoms during pregnancy. In comparison, only 3 studies (3.8%) focused on other aspects of women's reproductive mental well-being, including 2 on menopause-related mental well-being and 1 on menstrual-related mental well-being.

Furthermore, a small but noticeable portion of studies (9 studies, 11.7%) focused on specific mental well-being challenges faced by marginalized women, such as mental health for female refugees (Zaman et al) and the psychological well-being of unmarried teenage mothers in rural Uganda (Leerlooijer et al., 2014). The full list of these studies is in Table 3, along with the demographics analysis section.

Another prevalent category (10.1%) is interventions aimed at healthy lifestyles and dietary issues, focusing on tackling eating disorders and encouraging physical activity to support holistic well-being for women. Moreover, another prevention sector, “safety empowerment of women,” appeared in 8.8% of the studies. Most of these studies focused on women in India who face ongoing threats of violence, including physical or sexual abuse. Researchers primarily developed IoT-based wearable devices designed for safety and alert systems to address these concerns.

### 3.3 Indication of design methods adopted and user involvement

#### 3.3.1 Indication of design methods

**Fig. 5:**
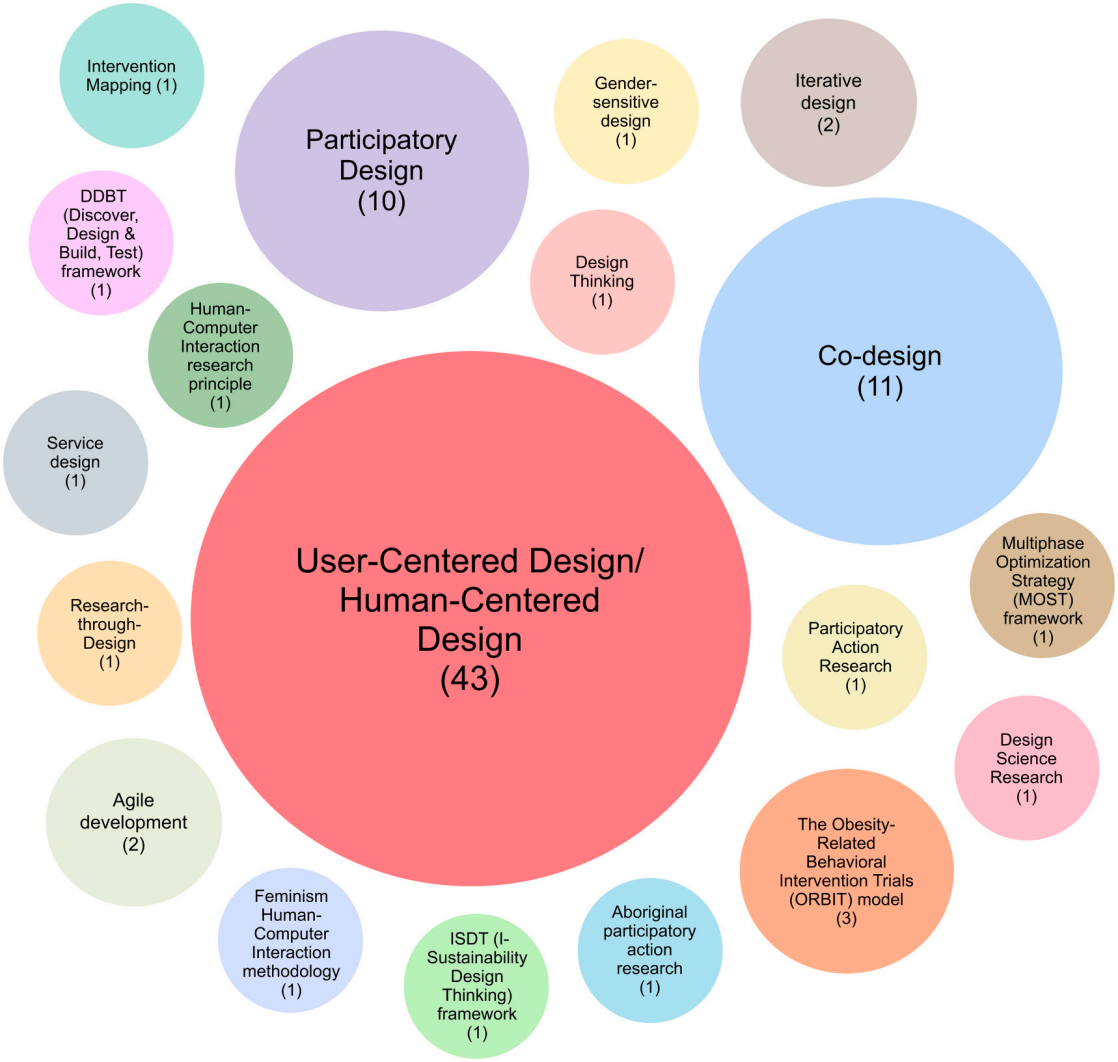
Types of design methods indicated.

To avoid ambiguity, by design methods we mean the methods that structure or guide the overall development of the process, rather than specific methods for design activities, e.g., usability testing or co-creation sessions. Given the interdisciplinary nature of digital mental health research, we adopted broad criteria when extracting design methods, including design frameworks and processes that guide the overall development of interventions. Fig. 5 illustrates the distribution of the mentioned design methods, and Table 2 introduces the definition and principles of each method.

**Table 2:** Definition of the principles of each design method mentioned in the studies.

Design methods	Definition and design principles
User-centered design/human-centered design	An iterative process that focuses on users and their needs at every stage of design, often involving user research, concept development, usability testing, and refinement to align solutions with real-world user behaviors (Norman, 1988; Rogers et al., 2011).
Participatory design	Involves end users (or co-designers) in critical decision-making and design activities from the outset, enabling their direct influence on the intervention's development and ensuring outputs reflect users' lived experiences (Schuler and Namioka, 2017).
Co-design	Emphasizes collaboration between diverse stakeholders, such as designers, researchers, and end-users, throughout the design process, fostering shared ownership and integrating a wide range of perspectives (Sanders and Stappers, 2008).
Design thinking	Adopts a human-centered, iterative approach to problem-solving by integrating empathy, ideation, prototyping, and testing to generate user-driven solutions. It emphasizes collaborative methods and rapid experimentation to tackle complex challenges (Razzouk and Shute, 2012).
The ORBIT model	A phased approach to developing behavioral interventions for obesity-related issues, beginning with basic research to define problems and intervention components, then moving through iterative pilot testing and larger-scale trials to refine the intervention for maximum effectiveness (Czajkowski et al., 2015).
Participatory action research	A cyclical, collaborative research approach where researchers and participants work together to identify problems, develop strategies, and implement changes, aiming to empower participants and stimulate community-led transformations (Chevalier and Buckles, 2019).
Aboriginal participatory action research	An adaptation of Participatory Action Research that prioritizes Indigenous knowledge, culture, and practices, ensuring community-led decision-making and cultural safety while respecting tribal sovereignty (Dudgeon et al., 2020).
Human-computer interaction principles	Centers on usability, accessibility, user satisfaction, and overall experience by prioritizing consistency, providing clear feedback, implementing strong error handling, and conducting iterative testing to ensure the system design meets user needs (Dix, 2003).
Service design	Combines design thinking, systems thinking, and user-centered methods to enhance or develop services by examining all touchpoints in the service ecosystem, with the goal of improving user experiences, stakeholder satisfaction, and operational efficiency (Stickdorn and Schneider, 2012).
I-sustainability design thinking framework	Merges sustainability values with design thinking methods, incorporating iterative prototyping, stakeholder collaboration, and long-term environmental considerations to balance immediate user needs with global ecological objectives (Chiou et al., 2021).
Feminism human-computer interaction methodology	A methodology proposed by HCI scholars (Bardzell and Bardzell, 2011) that integrates feminist theory and social science in HCI research. It advocates seven key methodological positions: simultaneous commitment to scientific and moral objectivities, connection to feminist theory, commitment to methodology, empathetic relationship with participants, researcher/practitioner self-disclosure, co-construction of the research, and the adoption of diverse and/or mixed methods.
Gender-sensitive design	A design methodology that treats gender as a critical analytical variable throughout the design process, challenging the assumption of a neutral “default” user (Schiebinger, 2014). It calls for systematic gender analysis at each design stage. Core practices include sex-disaggregated data collection, diversity personas, and participatory methods that actively involve users of different genders in co-creating design outcomes (Ballesteros et al., 2025).
Research-through-design (RtD)	A methodology that uses design experiments to generate knowledge and insights within a research context (Stappers and Giaccardi, 2017). Unlike traditional experiments in the social sciences where a hypothesis is tested under controlled conditions, design experiments in RtD are often open-ended and aim to explore new ideas and solutions (Stappers and Giaccardi, 2017; Zimmerman et al., 2007).
Agile development	A software development methodology prioritizing iterative delivery over rigid upfront planning, formalized in the Manifesto for Agile Software Development by seventeen practitioners (Beck et al., 2000). It centers on four core values: individuals and interactions, working software, customer collaboration, and responding to change.
DDBT (discover, design & build, test) framework	By adopting HCD methods, the DDBT framework treats usability as an implementation determinant and guides iterative redesign of clinical interventions and implementation strategies (Lyon et al., 2019). It structures design into three phases: Discover (identify usability barriers), Design/Build (co-design solutions with users), and Test (evaluate and refine).
Multiphase optimization strategy (MOST) framework	An engineering-inspired framework for developing, optimizing, and evaluating multicomponent behavioral interventions, developed by Collins et al. (2007). MOST emphasizes the systematic selection and refinement of intervention components through iterative experimentation before conducting a definitive randomized controlled trial. It structures intervention development into three phases: preparation, optimization, and evaluation.
Iterative design	An approach that develops a solution through repeated cycles of prototyping, testing, and refinement, using user or stakeholder feedback to improve the design over time (Gould and Lewis, 1983; Nielsen, 1993). It is a core element underlying many design methods, including HCD/UCD, Design Thinking, and Agile Development.
Design science research	A research methodology originating in the field of Information Systems that develops and evaluates innovative artifacts, such as systems or models, to solve identified real-world problems. It combines iterative design, implementation, and evaluation to generate both practical solutions and scientific knowledge (Hevner et al., 2004).
Intervention mapping	A systematic framework for designing theory- and evidence-based health interventions in health promotion. It guides developers through a series of steps, including needs assessment, defining intervention objectives, selecting theory-informed strategies, developing intervention components, planning implementation, and evaluation (Bartholomew Eldredge et al., 2016).

Firstly, 18 out of 77 studies (22.8%) did not explicitly state which design methods were used to guide the development of DMHIs. Of these, 8 focused on technology development of the interventions (6 developed wearable devices, 2 developed mobile apps), but did not report the design process or methods (Saravanan et al., 2022; Mahajan et al., 2016; Economou et al., 2017; Hossain et al., 2020; Savithri et al., 2023; Vikruthi et al., 2023; Chandra et al., 2023; Mumbaikar et al., 2025); 2 studies primarily tested the intervention for clinical effectiveness rather than the design process (Fealy et al., 2019; Jiang et al., 2024); and 3 studies mentioned that the intervention was designed by the research team or experts, without specifying the adopted design methods (Economou et al., 2017; Gewali et al., 2021; McCall et al., 2021). Moreover, among these 21 studies, 3 (Senette et al., 2023; Amore et al., 2023; D, Bhagat and S, 2025)) reported user involvement in their design process but did not specify the design methods they used.

Secondly, among the 60 (77.2%) studies that reported at least one design method, 19 different methods were identified (as shown in Fig. 5). Human-Centered Design (HCD)/User-Centered Design (UCD) was the most common (71.2%), followed by co-design (13.9%) and Participatory Design (12.7%). Eighteen studies reported using multiple design methods, such as combining UCD and co-design. Among the less frequently reported approaches were several emerging and specialized frameworks, including the ISDT (I-Sustainability Design Thinking) framework, originally developed within sustainability research, and the Obesity-Related Behavioral Intervention Trials (ORBIT) model, which combines behavioral theory with UCD principles. Additionally, two studies published in 2025 reported adopting feminism-oriented design approaches: Anwar and Koenitz (2025) used Feminism Human-Computer Interaction (HCI) methodology and entailed how it guided their research; and Subashini M et al. (2025) reported adopting Gender-Sensitive Design, although the method was not explicitly defined or referenced in their study. A complete overview of the studies associated with each design method is provided in Appendix B, Supplementary Table 2.

Nevertheless, among the 59 studies that reported adopting design methods, 8 provided little detail on how these methods specifically informed the design process (Perego and Terraroli, 2023; Pereira et al., 2026; Rodriguez and Jareda, 2019; Apsey et al., 2024; Elwahsh et al., 2025; Daniels et al., 2024; Nicoletti et al., 2025; Subashini et al., 2025). This makes it hard to assess how consistently these methods were adopted. That is, they did not specify or reference which principles or aspects from this method guided their research, or which activities (e.g., persona development/journey mapping under HCD/UCD, user advisory board under PD) were carried out using each method.

#### 3.3.2 User involvement across design stages and activities

**Fig. 6:**
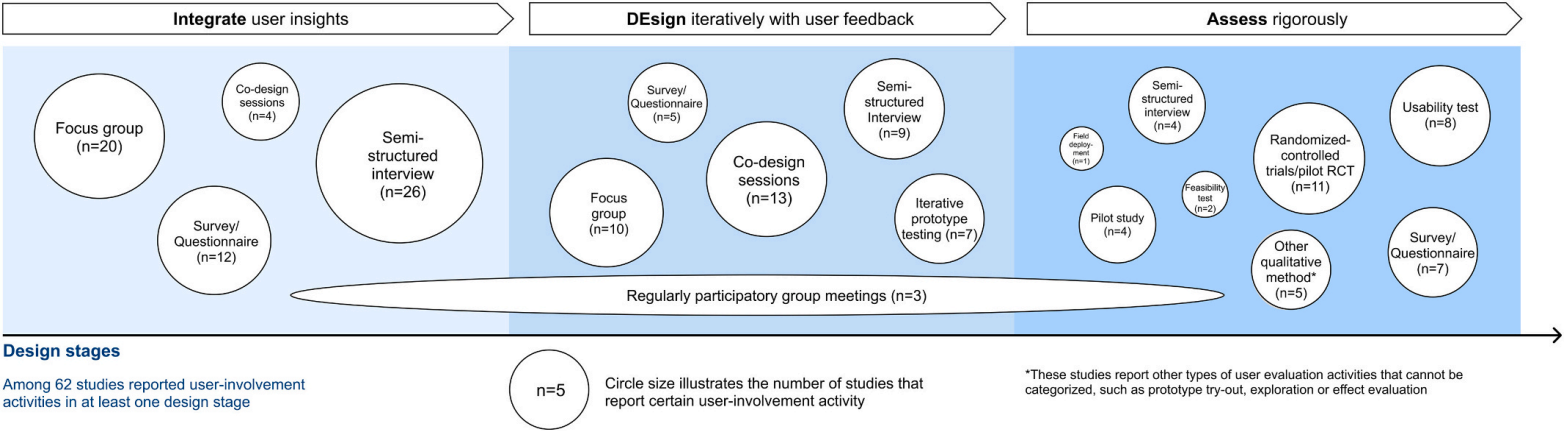
User-involved activities and their counts across design stag0000

Fig. 6 shows an overview of user involvement across the three design stages identified by the IDEAS process (Mummah et al., 2016), based on the 62 (80.5%) studies that reported user involvement activities in at least one design stage, and summarizes the prevalent activities conducted at each stage. A synthesized table of the coded user involvement activities and corresponding design stages for each included study is provided in Appendix B, Supplementary Table 3.

In the Integrate phase, the semi-structured interview is the most reported activity, mentioned by 26 studies. Furthermore, the co-design session is most prominent in the Design phase (*n* = 13), while in the Assess phase, Randomized Controlled Trial (RCT)/Pilot RCT is most mentioned (*n* = 12). To minimize bias, we examined related publications linked to the included studies to identify any additional reports of user involvement activities. When such activities were reported in related articles, they were also counted as instances of user involvement.

15 out of 77 (19.5%) studies showed no evidence of user involvement activities. Among them, two studies reported using participatory design or HCD/UCD but did not specify any user involvement activities (Sreeraj et al., 2020; Pereira et al., 2026). Additionally, five studies clearly mentioned the involvement and roles of specific stakeholders during the development of the intervention (Economou et al., 2017; Fealy et al., 2019; Chiou et al., 2021; Sreeraj et al., 2020; Ahmed et al., 2024).

Out of the 62 studies that reported user involvement, 38 reached the Assess phase. However, 17 of these (44.7%) did not report activities from one or both of the earlier stages, Integrate and Design. Among these 17 studies, user involvement in the Design phase was most often omitted (10 studies, 58.8%), whereas 6 studies did not report user involvement in the Integrate phase. Additionally, three studies involved users only during the Assess stage, such as through usability testing or randomized controlled trials (RCTs), and collected feedback after the intervention was designed via user interviews or focus groups.

In contrast, 16 out of 77 (20.8%) studies reported full user involvement throughout the stages, including activities such as early-stage user interviews, focus groups, co-design workshops, user testing, and Randomized Controlled Trials (RCTs). These studies involved users through various user-engaged activities throughout different stages. For instance, Gordon et al. (2015) established user-involved, multidisciplinary groups. They organized regular meetings and design activities with end-users, which included them in decision-making from the earliest stages and throughout development.

### 3.4 Demographic characteristics of involved users

The demographic data of involved users were challenging to categorize due to a pervasive lack of data on user demographics and inconsistent, varied approaches to reporting demographic data across the included studies. A synthesized table of demographic characteristics is provided in Appendix B, Supplementary Table 4.

#### 3.4.1 Age

Age information was inconsistently reported across the included studies. More than half of the studies (39/77, 50.6%) did not report participants' ages. Among the 38 studies that provided age data, reporting formats varied, including means, age ranges, and age group distributions. We categorized them into four groups: Adolescents (<18); Reproductive-age women (18–44); Perimenopausal (45–55); Postmenopausal (>55). Of these, one study targeted teenage mothers aged 15–19; 26 of 38 (68.4%) studies focused on reproductive-age women (18–44); three targeted perimenopausal women (40–55); and one focused on postmenopausal women (>55). 7 studies included broad age ranges spanning multiple life stages, with 4 spanning both reproductive and perimenopausal stages, and 3 spanning reproductive, perimenopausal, and postmenopausal stages.

#### 3.4.2 Education level

A significant portion, 67.5% of studies (52 studies), did not report the education level of their involved users. Meanwhile, 32.5% of studies (25 studies) reported the education-level composition of users, with corresponding percentages. Among these, 69.2% of their involved users had college or university education or higher (including those who may not have completed a degree); 25.4% had secondary education (some or complete high school or secondary school); and a small percentage, 5.4%, had below-secondary education.

Moreover, 4 studies reported education levels in other forms, such as technical degrees and mean years of education. Therefore, we did not include them in the calculation of educational level composition.

#### 3.4.3 Annual income

Most studies (67 studies, 87.0%) did not report their users' annual income. The remaining studies provided income information in various ways, including whether users had a history of poverty or housing insecurity, whether they were employed, and by income bracket.

#### 3.4.4 Race/ethnicity and immigration status

64.9% of studies (50 studies) did not report the race/ethnicity or immigration status of their participants. In 9.1% of studies (7 studies), users with minority backgrounds, such as Latino/Black women, Indigenous women, and women with immigration backgrounds, were most prominent.

#### 3.4.5 Studies targeting marginalized groups

9 studies (11.7%) specifically targeted well-being challenges of marginalized women, including those from minority racial or ethnic groups, low socio-economic backgrounds, or migration backgrounds. Table 3 provides an overview of these studies, highlighting the populations they served. Notably, many of them also adopted the HCD process, which consistently involves end-users and community-based methods, such as Aboriginal participatory action research (Zaman et al., 2019; Perkes et al., 2022, Perkes et al., 2023).

**Table 3:** Studies target women from marginalized groups.

Type of mental well-being problem of marginalized women	Study identifier
Mental health condition of the female refugees in Bangladesh	Zaman et al., 2019
Psychological and social well-being of unmarried teenage mothers in rural Uganda	Leerlooijer et al., 2014
Health challenges of low-income Caribbean transmigrant women	AlJaberi, 2018a, AlJaberi, 2018b
Build social cohesion to increase physical activity for low-resourced mothers in urban settings.	Houghton et al., 2021
Empowerment among women from rural communities in India	Sreeraj et al., 2020
Well-being of Aboriginal and Torres Strait Islander Mothers	Perkes et al., 2022, Perkes et al., 2023
Empowerment of rural women in India	Menon, Nampoothiri, F, B and Rao, 2024
Enhance bodily awareness and emotional well-being among Afghan women facing restrictive cultural norms	Anwar and Koenitz, 2025
Stress related to serostatus disclosure for transgender women in Africa	Daniels et al., 2024

#### 3.4.6 Consideration of intersecting identities

33.8% of the studies (26 studies) reported that women involved in the design process possessed at least two intersecting cultural identities across race/ethnicity, socioeconomic status, immigration status, language, sexuality, and gender orientation. These studies included considerations of multiple cultural identities when selecting target users, applying an intersectional lens to understand and analyze user experiences, designing intervention features that address intersecting identities, and evaluating intervention outcomes based on these identities.

Firstly, most of these studies (23/26) took intersecting identities into account when choosing their target groups, such as designing DMHI specifically for indigenous women or women from minoritized racial/ethnic backgrounds (Zaman et al., 2019; Singleton et al., 2021, Singleton et al., 2023; Gordon et al., 2015; AlJaberi, 2018a, AlJaberi, 2018b; Houghton et al., 2021; Ashmore et al., 2020; Fealy et al., 2019; McCall et al., 2021; Figueroa et al., 2021b; Antelo et al., 2021; Rau et al., 2020; Perkes et al., 2022, Perkes et al., 2023; Gilbert et al., 2023a, Gilbert, Irvine, D'Or, Rae and Murphy, 2023b; Bhat et al., 2023; Reen and Orji, 2022; Menon et al., 2024; Zimmermann et al., 2025; Ardi et al., 2025; De Bruin et al., 2026; Anwar and Koenitz, 2025; Horowitz et al., 2025; Gewali et al., 2021; Elwahsh et al., 2025; Ahmed et al., 2024; Daniels et al., 2024; Mustafina et al., 2025).

Secondly, 18 studies reported activities aimed at understanding and analyzing women's experiences related to intersecting cultural identities (Barry et al., 2017; Gordon et al., 2015; AlJaberi, 2018a, AlJaberi, 2018b; Houghton et al., 2021; McCall et al., 2021; Figueroa et al., 2021b; Perkes et al., 2022, Perkes et al., 2023; Gilbert et al., 2023a, Gilbert, Irvine, D'Or, Rae and Murphy, 2023b; Bhat et al., 2023; Menon et al., 2024; Zimmermann et al., 2025; De Bruin et al., 2026; Anwar and Koenitz, 2025; Sugarman et al., 2026; Gewali et al., 2021; Elwahsh et al., 2025; Ahmed et al., 2024; Daniels et al., 2024). For example, Zaman et al. (2019) examined the specific mental well-being needs of female refugees using DMHI, and Perkes et al., 2022, Perkes et al., 2023 explored the cultural and community-specific needs of Aboriginal and Torres Strait Islander women and children.

Moreover, 12 studies incorporated specific functions or design features in the DMHI that address users' identified intersectionality needs (Gordon et al., 2015; Figueroa et al., 2021b; Perkes et al., 2022, Perkes et al., 2023; Bhat et al., 2023; Zimmermann et al., 2025; Ardi et al., 2025; De Bruin et al., 2026; Anwar and Koenitz, 2025; Sugarman et al., 2026; Elwahsh et al., 2025; Ahmed et al., 2024; Daniels et al., 2024). For example, Anwar and Koenitz (2025) adopted symbolic metaphors to convey information when designing a mental health app for Afghan women, allowing users to safely engage with the app on their devices without fear of discovery or surveillance.

Additionally, only one study (Figueroa et al., 2021b) assessed differences in intervention outcomes across diverse cultural identities, comparing outcomes between English-speaking and Spanish-speaking users.

## 4 Discussion

### 4.1 Gaps and challenges

The review highlights several significant challenges related to adopting design methods, the level of user involvement, and the inclusion of diversity and inclusivity in the development of DMHIs for women. Overall, we found that many studies in our review do not report on the design methods used, the activities performed with these methods, or the characteristics of the population involved. When users were involved, they were mostly only partially involved in one or two design phases, rather than consistently involved throughout the design process. Additionally, there is a noticeable overrepresentation of research from higher-income countries and an underrepresentation of women from racial or ethnic minority backgrounds, those with lower education levels, and older women. This limits DMHI's accessibility and effectiveness for all women and diminishes its potential to promote diversity, inclusivity, and ultimately, health equity.

#### 4.1.1 Inconsistent application and reporting of design methods

A significant number of studies did not clearly indicate which design methods they adopted. In addition, among studies that described the use of design methods such as HCD and PD, the methodological descriptions were superficial, with many studies not reporting the steps undertaken. This suggests a gap between the stated use of user-centered methods and their comprehensive application across all phases of DMHI development, or a gap in the reporting of the user-centered methods. Our findings align with earlier research (a systematic review of 30 studies until 2022) in digital mental health (not specific to women), in which authors described that, though some attempts have been made to integrate human-centered design approaches into DMHI development, they rely very little on designers and design research (Vial et al., 2022). Here, specifically for the women's DMHI field, we show that, until 2026, human-centered design approaches are still not applied as intended and are instead superficially reported.

Notably, very few studies reported activities corresponding to the Share phase of the IDEAS framework, such as dissemination, implementation, or long-term scaling strategies. This limited reporting may reflect broader challenges in applying the full IDEAS framework in practice, particularly within academic health research settings that often prioritize intervention development and short-term evaluation over implementation and sustainability (Graham et al., 2020; Iyamu et al., 2022). Existing implementation science literature has similarly highlighted that digital health interventions frequently face difficulties related to long-term integration, dissemination, sustainability, and translation into routine practice, especially within resource-constrained or interdisciplinary health research environments (McCool et al., 2020; Graham et al., 2020). It may also indicate that dissemination and scaling activities remain insufficiently documented in DMHI research.

Our findings could have several reasons. First, a potential reason for the superficial adoption of HCD methods is that funding organizations or publishers often encourage their inclusion without providing sufficient resources, guidance, or oversight to ensure robust implementation (Kilbourne et al., 2007). This could lead to the “check-box mentality” that researchers tend to fulfill these expectations simply by organizing one-time user sessions or just citing HCD in their methodology.

Moreover, the misalignment between the design sciences and traditional academic structures in mental health research may contribute to this gap. HCD emerged from the design field, which involves rapid prototyping and continuous iteration based on user feedback, while most academic health studies follow pre-established protocols and strict timelines. Specifically, medical sciences often prioritize controlled trials, which are typically conducted after an intervention has already been developed. Consequently, if time and resources are to be allocated to user involvement, this tends to occur post-development, during the recruitment phase for randomized controlled trials (RCTs). Consequently, HCD activities may be compromised to fit the medical disciplines' research cycle. Another factor is the lack of HCD expertise among mental health research teams. One possible explanation is that some mental health researchers may have limited familiarity with design thinking or user-centered methods, which could contribute to challenges in implementing HCD throughout the intervention development process.

To address these challenges, we advocate collective efforts between the digital health research teams, designers, policymakers, and funding agencies. Research teams should adopt multidisciplinary collaborations involving design researchers from the earliest stages of development. Funding agencies could provide detailed guidelines for iterative and participatory processes as a condition for project approval or continued funding. Moreover, app assessment frameworks should offer clearer standards for user involvement. While current frameworks, such as the ORCHA-based frameworks and assessment tools, acknowledge the importance of user involvement and evaluate aspects of usability and user experience, with little emphasis on the design process or how users were involved during development (Frey et al., 2023; Weirauch et al., 2024). Lastly, as interventions need to be iteratively improved, so must design methods. Designers should enhance current HCD methods or develop new ones that meet the specific demands of digital health research, and this requires mutual learning across both the design and health domains (Stiles-Shields et al., 2022).

#### 4.1.2 Lack of indication of key design deliverables

Another recurring challenge in the reviewed studies is the ambiguity about whether an intervention has reached the minimum viable product (MVP) stage. While many studies mentioned iterative prototyping or product assessment, it was often unclear whether they were still refining core features (Design stage) or had already moved on to evaluating a stable MVP (Assess stage). Methodologically, this distinction is important to clarify. Iterative testing and refinement usually occur before an MVP is finalized, when user feedback can meaningfully influence the interface, features, or user experience. In contrast, once a product is considered “viable,” subsequent tests tend to focus more on effectiveness or usability rather than major reconfiguration. This lack of clarity makes it difficult to determine how user insights affected design decisions and the extent to which researchers integrated iterative feedback into their product. Future studies should clearly indicate the product's status (e.g., prototype versus MVP) and explain how user feedback shaped its development.

#### 4.1.3 Lack of consideration of diversity, inclusivity, and intersectionality

The results also highlight a significant gap in addressing diversity, inclusivity, and intersectionality when developing DMHIs for women. For example, studies mainly focus on women from high-income, developed countries, while women from developing nations, minoritized racial or ethnic groups, or immigrant backgrounds are underrepresented. This reveals a failure to adequately consider the diverse identities of women in current DMHIs.

Additionally, we noted a lack of consideration for women's intersecting identities during both study scoping and data analysis. This is important because exploring intersectional differences in needs related to digital health tools, uptake, and outcomes can help customize DMHIs more effectively for specific population subgroups and prevent the design of interventions that do not work for anyone by ignoring heterogeneity (Figueroa et al., 2021a).

Lastly, good practices originate from a small but promising number of studies that explicitly target mental health challenges among marginalized women and address their unique needs related to cultural identities. These studies mainly focus on Indigenous women, women from rural areas in developing countries, or minoritized racial and ethnic groups. For example, Perkes et al., 2022, Perkes et al., 2023 investigated and addressed the specific mental well-being needs of Aboriginal and Torres Strait Islander women through iterative co-design process. The app content highlighted culturally relevant health information tailored to users' health literacy levels. Notably, this study also assessed users' perceptions of the app's cultural relevance. Similarly, Menon et al. (2024) developed a seasonal calendar app for rural women in India. This app includes users' regional seasonal markers and native language (Malayalam), aligning with their linguistic and cultural context. Furthermore, Gilbert et al., 2023a, Gilbert, Irvine, D'Or, Rae and Murphy, 2023b used community-based co-design when creating an app for Indigenous women, and the intervention is explicitly tailored for urban, regional, and remote Indigenous communities, taking into account digital access and local literacy or language needs. Interestingly, these studies that include marginalized women often do a better job of reporting and applying HCD methods.

### 4.2 Proposed research agenda

*Suggested research agenda for better reporting and using design methods for researchers*:

(1) Explain the rationale and principles behind the selected design method: Rather than only naming the chosen design methods (such as “we followed the principles of User-Centered Design”), researchers should describe how the method guides each phase of their work in the development of DMHIs. Specifically, researchers should indicate which design principles are followed, how they were integrated into the study, and reference the relevant literature or guidelines of the chosen method.
(2) Coherently apply HCD methods and engage users at each stage of DMHI development: After systematically structuring the process of DMHI development based on chosen design methods, researchers should strive to adhere coherently to the design principles and involve end users from the earliest ideation through to the final evaluation.
(3) Clearly document each design stage and use precise terminology. To improve the clarity of user involvement at different design stages of the development of DMHIs, it is helpful to name each stage explicitly and note which stages have been completed. If certain phases are part of a different publication or an ongoing effort, researchers should specify this. Moreover, Researchers often use terms like “iterative testing” or “product assessment” without clarifying whether they are refining features in a prototype (Design stage) or evaluating a minimum viable product (Assessment stage). Because these testing activities differ in scope and objectives, authors should explicitly state which type of testing is conducted, the level of user involvement, and the specific implications for subsequent design decisions.
(4) Clearly document the demographics information of participants: Specify the demographics information, including age, ethnicity, etc., of the recruited participants of user-involved activities. Reflect on whether the design process considered intersectional identities. This leads to research that is more generalizable to a wider population. It also enables researchers to reflect on their process in the future.
(5) Keep track of the interaction and power balance during user-involved activities:
  One reviewed study (Franco, Olhaberry, Kelders and Muzard, 2023, Franco, Olhaberry, Kelders, Muzard and Cuijpers, 2024a, Franco, Olhaberry, Muzard, Harismendy and Kelders, 2024) reports in their study, that during a focus group session with a mixed group of healthcare professionals and patients, the dominance of healthcare professionals overshadowed the opinions of the target users, and users were hesitant to share their perspectives. After the researchers observed this imbalance, they promptly adjusted the follow-up focus group settings and separated the two groups into different sessions. This highlights the importance of carefully shaping the power structure in the user-involved activities.

### 4.3 Limitations of this study

Though we followed a rigorous methodology and systematically applied our search strategies, it is possible that some relevant studies were not captured due to the use of different keywords in the literature or because certain studies are published in languages other than English, which were beyond the scope of our review. Additionally, while we limited the scope of intersectionality in this study by examining intersecting identities, more enablers of intersectionality theory, such as reflexivity and relational power (UN Women, 2022), have not yet been examined. Future research would benefit from adopting a more extensive intersectional lens that systematically examines these different enablers.

## 5 Conclusion

Our review reveals a persistent gap in the design and development of DMHIs for women, marked by superficial reporting of methods used in the design process, limited user involvement, and an underrepresentation of women from racial/ethnic minority backgrounds or low socioeconomic status. DMHIs hold great promises for supporting women's mental well-being. However, they currently fall short in serving women from diverse backgrounds as HCD processes are applied superficially, and an intersectional perspective is missing. As future priorities, researchers need to engage diverse populations of women and embed an intersectional lens to understand their specific mental health needs and experiences. At the same time, the application and reporting of HCD practices must be improved. To achieve this, we call for researchers from both design and health fields to work closely together in developing DMHIs by adapting current HCD methods with intersectionality theory, creating clear guidelines for their use and reporting, and embedding diverse cultural identities of users. In doing so, DMHIs can be better aligned with the realities of diverse women's lives, and ultimately promoting health equity.

## Declaration of Generative AI and AI-assisted technologies in the writing process

During the preparation of this work, the first author used ChatGPT to proofread academic writing and improve readability. After using this tool/service, the author reviewed and edited the content as needed and takes full responsibility for the content of the publication.

## Declaration of competing interest

The authors declare that they have no known competing financial interests or personal relationships that could have appeared to influence the work reported in this paper.

## Data Availability

Data described in the manuscript, code book, and analytic codes will be made available upon request pending [application and approval].

## Appendix A Supplementary data

Appendix A PRISMA checklist.

Appendix B Supplementary Table 1 Full search terms.

Appendix B Supplementary Table 2 Summary of studies associated with each design method.

Appendix B Supplementary Table 3 Coding of user involvement activities of the studies.

Appendix B Supplementary Table 4 Overview of demographics data extracted from the included studies (*N* = 77).
