# Supplementary material for: Designing for all: A scoping review and critical analysis on how digital mental health interventions serve (or fail) diverse women
Source: Internet Interv. 2026 Jul 16;45:100981. doi: 10.1016/j.invent.2026.100981 (PMC13448905; doi:10.1016/j.invent.2026.100981)
Supplement: Supplementary file 2 [file mmc2.docx]

| **Concept** | **Search Terms** |
| --- | --- |
| Mental well-being | (“Mental health” OR “Well-being” OR “Mental disease” OR “mental illness” OR “Mental disorder” OR “Psychiatry” OR “Depression” OR “Anxiety” OR “Bipolar” OR ”Abuse” OR “Alcohol” OR “Drug taking” OR "psychological wellbeing" OR "emotional wellbeing" OR "mental distress" OR "psychological distress" OR "mental illness" OR "mental disorder*" OR "psychiatric disorder*" OR "psychopathology" OR "psychological symptom*" OR "bipolar disorder" OR "PTSD" OR "post-traumatic stress" OR "eating disorder*" OR "addiction" OR "stress" OR "burnout" OR "resilience" OR "trauma" OR "poverty" OR "violence" OR "coping strategies") |
|  | AND |
| Women | (“female*” OR “women” OR “feminine” OR “girls” OR “pregnant” OR “pregnancy” OR “maternal” OR “Postpartum” OR “Abortion” “female-identifying” OR “trans women” OR “adolescent girls” OR “young women” OR “older women” OR “mother” OR “motherhood” OR "miscarriage" OR "gynaecological health" OR "women's health" OR "underserved women" OR "rural women" OR "Black women" OR "women of color" OR "Asian women" OR "Latina women" OR "immigrant women" OR "unemployed women" OR “perinatal” OR “peripartum” OR “menstruation” OR “menstrual health” OR “menstrual well-being” OR “menopause” OR “menopausal health” OR “menopause well-being” OR “perimenopause” OR “post menopause” OR “women’s health”) |
|  | AND |
| Design process | (“Design methods” OR “Design frameworks” OR “Design process” OR “Design thinking” OR “User-Centered Design” OR “Human-Centered Design” OR “Interaction Design” OR “User Experience (UX) Design” OR “User Interface Design” OR “UI design” OR “Participatory Design” OR “Co-design” OR "design strategy" OR "design-based research" OR "service design" OR "inclusive design" OR "universal design" OR "user-driven design" OR "needs-based design" OR "design workshops" OR "community-based design" OR "collaborative design" OR "person-centred design" OR "person-based approach" OR "UX research" OR "UI development" OR "interface prototyping" OR "iterative design" OR "prototyping process" OR "design iteration") |
|  | AND |
| Digital Interventions | (“Digital Health Interventions” OR “eHealth” OR “mobile health” OR “Wearable Devices” OR “Remote Patient Monitoring” OR “Telehealth” OR “Telemedicine” OR “Digital Therapy” OR “Application” OR “App” OR “Chatbot” OR "mHealth" OR "e-therapy" OR "digital mental health" OR "technology-based intervention*" OR "smartphone*" OR "web-based intervention*" OR "online program*" OR "self-guided intervention*" OR "digital CBT" OR "digital self-help" OR "mobile-based therapy" OR "digital mental health platform*") |

Supplementary Table 1: Full search terms
