# Supplementary material for: Designing for all: A scoping review and critical analysis on how digital mental health interventions serve (or fail) diverse women
Source: Internet Interv. 2026 Jul 16;45:100981. doi: 10.1016/j.invent.2026.100981 (PMC13448905; doi:10.1016/j.invent.2026.100981)
Supplement: Supplementary file 3 [file mmc3.docx]

**Supplementary Table 2: Summary of studies associated with each design method**

| **Design method adopted** | **Identifiers of associated studies** |
| --- | --- |
| No clear indication of design methods adopted | Saravanan et al., 2022; Mahajan et al., 2016; Hossain et al., 2020; Savithri et al., 2023; Economou et al., 2017; Vikruthi et al., 2023; Tamanna et al., 2021; Chandra et al., 2023; Senette et al., 2023; Osman et al., 2023; Moshki et al., 2014; Fealy et al., 2019; McCall et al., 2021; Sun et al., 2021; Dharani et al., 2025; Mumbaikar et al., 2025; Gewali et al., 2021; Jiang et al., 2024 |
| HCD/UCD | Dauber et al., 2022; Isaacs et al., 2023; Barry et al., 2017; Antelo et al., 2022; Rau et al., 2020; Sreeraj et al., 2020; Lackie et al., 2021; Singleton et al., 2021; Eaves et al., 2023; Amore et al., 2023; Perego et al., 2023; Gill et al., 2024; Bhat et al., 2023; Reen et al., 2022; Mancinelli et al., 2024; Franco et al., 2024; Fietta et al., 2024; Menon et al., 2024; Materia et al., 2018; Rizzi et al., 2025; McAlister et al., 2025; et al., Zimmermann 2025; Subashini et al., 2025; Ardi et al., 2025; de Bruin et al., 2026; Nicoletti et al., 2026; Pereira et al., 2026; Horowitz et al., 2026; Pourshahrokhi et al., 2026; Sugarman 2026; Nuseibeh et al., 2024; Apsey et al., 2024; Elwahsh et al., 2025; Wang et al., 2025; Liu et al., 2021; Daniels et al., 2025; Nicoletti et al., 2025; Shifflett et al., 2025; Osweiler et al. et al., 2025 |
| Participatory Design | Leerlooijer et al., 2014; Ashmore et al., 2020; Rau et al., 2020; Duffett-Leger et al., 2020; Sreeraj et al., 2020; Bhat et al., 2023; Horowitz et al., 2026; Rodríguez et al., 2019; Elwahsh et al., 2025 |
| Co-design | Singleton et al., 2023; Aljaberi et al., 2018; Houghton et al., 2021; Figueroa et al., 2021; Duffett-Leger et al., 2020; Singleton et al., 2021; Perkes et al., 2023; McKellar et al., 2023; de Bruin et al., 2026; Nicoletti et al., 2026; Nuseibeh et al., 2024 |
| Design thinking | Adrian et al., 2024 |
| The ORBIT model | Mancinelli et al., 2024; Rizzi et al., 2025; Nicoletti et al., 2026 |
| Agile development | Subashini et al., 2025; Horowitz et al., 2026 |
| Iterative Design | Ahmed et al., 2024; Daniels et al., 2025 |
| Participatory Action Research | Gordon et al., 2016 |
| Aboriginal Participatory Action Research | Gilbert et al., 2023 |
| Human-computer interaction principles | Zaman et al., 2019 |
| Service Design | Fietta et al., 2024 |
| ISDT framework | Chiou et al., 2021 |
| Research-through-Design | Anwar et al., 2025 |
| Gender Sensitive Design | Subashini et al., 2025 |
| Feminist Human-Computer Interaction (HCI) frameworks | Anwar et al., 2025 |
| DDBT (Discover, Design & Build, Test) framework | Sugarman et al., 2026 |
| Multiphase Optimization Strategy (MOST) framework | Dauber et al., 2022 |
| Iterative Design | Ahmed et al., 2024; Daniels et al., 2025 |
| Design Science Research (DSR) approach | Mustafina et al., 2025 |
| Intervention Mapping | Scott et al., 2022 |

**References for all included studies (including related longitudinal study publications) in this review:**

1. Ahmed, I., Mohtasim, S. N., Arpita, F. O., Islam, A., & Amin, M. A. (2024). A Conceptual Design Framework of GorbhoShongi App for Mental Well-Being Among Bangladeshi Pregnant and Postpartum Women. *Communications in Computer and Information Science*, 3–13. https://doi.org/10.1007/978-3-031-78561-0_1
2. AlJaberi, H. (2018a). Social Interaction Needs and Entertainment Approaches to Pregnancy Well-Being in MHealth Technology Design for Low-Income Transmigrant Women: Qualitative Codesign Study. *JMIR Mhealth and Uhealth*, *6*(4), e61. https://doi.org/10.2196/mhealth.7708
3. AlJaberi, H. (2018b). Developing culturally sensitive MHealth apps for Caribbean immigrant women to use during pregnancy: Focus group study. *JMIR Human Factors*, *5*(4), e29. https://doi.org/10.2196/humanfactors.9787
4. Amore, A. D., Britt, A., Alvarez, S. J. A., & Greenleaf, M. N. (2023). A Web-Based Intervention to Address Risk Factors for Maternal Morbidity and Mortality (MAMA LOVE): Development and Evaluation study. *JMIR Pediatrics and Parenting*, *6*, e44615. https://doi.org/10.2196/44615
5. Antelo, V. S., Szwarc, L., Paolino, M., Saimovici, D., Massaccesi, S., Viswanath, K., & Arrossi, S. (2021). A counseling mobile app to reduce the psychosocial impact of human papillomavirus testing: Formative Research using a User-Centered Design approach in a Low-Middle-Income setting in Argentina. *JMIR Formative Research*, *6*(1), e32610. https://doi.org/10.2196/32610
6. Anwar, P., & Koenitz, H. (2025). A culturally sensitive interactive digital narrative to promote bodily awareness among Afghan women. In *Lecture notes in computer science* (pp. 147–170). https://doi.org/10.1007/978-3-032-12405-0_9
7. Apsey, C., Di Florio, A., & Stawarz, K. (2024). Developing a Mood and Menstrual Tracking App for People With Premenstrual Dysphoric Disorder: User-Centered Design Study. *JMIR Formative Research*, *8*, e59333. https://doi.org/10.2196/59333
8. Ardi, Z., Putra, A. H., Eseadi, C., & Sumari, M. B. (2025). GETO Application Development: A CulturallyGrounded gamified exposure therapy platform for managing anxiety in functional gastrointestinal disorder. In *2025 9th International Conference on Information, Control, and Communication Technologies (ICCT)* (pp. 1–4). https://doi.org/10.1109/icct67028.2025.11427432
9. Ashmore, L., Stewart, H., Hutton, D., & Evans, K. (2020). Digital support for living with and beyond gynaecological cancer. *Radiography*, *26*(4), e270–e276. https://doi.org/10.1016/j.radi.2020.03.014
10. Barry, M., Doherty, K., Belisario, J. M., Car, J., Morrison, C., & Doherty, G. (2017). mHealth for Maternal Mental Health. *Conference on Human Factors in Computing Systems - Proceedings 2017*. https://doi.org/10.1145/3025453.3025918
11. Bhat, A., Goud, B. R., Kalidindi, B., Ruben, J. P., Devadass, D., Waghmare, A., Collins, P. Y., Raj, T., & Srinivasan, K. (2023). Mobile Mental Health in Women’s Community-Based Organizations: Protocol for a pilot randomized controlled trial. *JMIR Research Protocols*, *12*, e42919. https://doi.org/10.2196/42919
12. Bhat, A., Goud, B. R., Pradeep, J. R., Jayaram, G., Radhakrishnan, R., & Srinivasan, K. (2020). Can mobile health improve depression treatment access and adherence among rural Indian women? a qualitative study. *Culture Medicine and Psychiatry*, *44*(4), 461–478. https://doi.org/10.1007/s11013-019-09664-3
13. Bhat, A., Johnson-Pradeep, R., Kalidindi, B., Devadass, D., Goud, B. R., Raj, T., Selvam, S., Navarro-Aguirre, Y., Collins, P. Y., & Srinivasan, K. (2025). Multiuser Application for the diagnosis and Treatment of Depression in Women’s Self-Help Groups: pilot randomized controlled trial. *JMIR Formative Research*, *9*, e68052. https://doi.org/10.2196/68052
14. Chandra, K. R., Nakka, T., Nadigatla, N., Reddy, N. V. S. T. S. V., Nulu, S., & Kali, A. (2023). Empowering Safety: Arduino-Based alert system design and implementation for women. *2020 4th International Conference on Electronics, Communication and Aerospace Technology (ICECA)*, 300–305. https://doi.org/10.1109/iceca58529.2023.10395065
15. Chiou, W., Hsu, S., Liang, Y., Hong, T., Lo, L., Chen, H., & Liu, C. (2021a). ISDT case study of We’ll app for postpartum depression women. In *Lecture notes in computer science* (pp. 119–137). https://doi.org/10.1007/978-3-030-77077-8_10
16. Chiou, W., Hsu, S., Liang, Y., Hong, T., Lo, L., Chen, H., & Liu, C. (2021b). ISDT Case Study of We’ll App for Postpartum Depression Women. In *Lecture notes in computer science* (pp. 119–137). https://doi.org/10.1007/978-3-030-77077-8_10
17. D, N., Bhagat, A., & S, D. C. (2025). Align: Empowering High School Students with PMS Awareness and Holistic Support. In *2025 7th International Congress on Human-Computer Interaction, Optimization and Robotic Applications (ICHORA)* (pp. 1–6). https://doi.org/10.1109/ichora65333.2025.11017097
18. Daniels, J., Van Der Merwe, L. L., Portle, S., Bongo, C., Nadkarni, S., & Peters, R. P. (2024). Tailoring a Skills-Based Serostatus Disclosure Intervention for Transgender Women in South Africa: Protocol for a Usability and Feasibility Study. *JMIR Research Protocols*, *14*, e52121. https://doi.org/10.2196/52121
19. Dauber, S., Barrie, K., Hogue, A., Henderson, C., Szmutko, E., Ford, V., Brown, J., Scott, L., & Ondersma, S. J. (2025). Feasibility and Acceptability of Integrating Digital Screening and Brief Intervention for Substance Use into Home Visiting: Lessons Learned from a Pilot Study. *Journal of Evidence-Based Social Work*, *23*(2), 303–327. https://doi.org/10.1080/26408066.2025.2536011
20. Dauber, S., Beacham, A., Hammond, C., West, A., & Thrul, J. (2022). Adaptive text messaging for postpartum risky drinking: Conceptual Model and Protocol for an Ecological Momentary Assessment study. *JMIR Research Protocols*, *11*(4), e36849. https://doi.org/10.2196/36849
21. Dauber, S., Hammond, C., Hogue, A., Henderson, C., Nugent, J., Ford, V., Brown, J., Scott, L., & Ondersma, S. (2022). Development of an electronic screening and brief intervention to address perinatal substance use in home visiting: Qualitative User-Centered Approach. *JMIR Formative Research*, *6*(11), e37865. https://doi.org/10.2196/37865
22. Dauber, S., West, A., Hammond, C., Cohen, I., & Thrul, J. (2021). Postpartum heavy episodic drinking: A survey to inform development of a text messaging intervention. *Drug and Alcohol Review*, *41*(1), 182–187. https://doi.org/10.1111/dar.13341
23. De Bruin, R. J., Figueroa, C. A., Broeke, P. T., De Jonge, K. N., Rousian, M., Steegers-Theunissen, R. P., & Rosman, A. N. (2026). Towards effective digital lifestyle interventions for pregnant women with obesity: A qualitative study exploring women’s and healthcare providers’ perspectives. *Digital Health*, *12*, 20552076251408518. https://doi.org/10.1177/20552076251408518
24. Doherty, K., Barry, M., Marcano-Belisario, J., Arnaud, B., Morrison, C., Car, J., & Doherty, G. (2018). A mobile App for the Self-Report of Psychological Well-Being During Pregnancy (BrightSelf): Qualitative Design study. *JMIR Mental Health*, *5*(4), e10007. https://doi.org/10.2196/10007
25. Eaves, E. R., Doerry, E., Lanzetta, S. A., Kruithoff, K. M., Negron, K., Dykman, K., Thoney, O., & Harper, C. C. (2022). Applying User-Centered design in the development of a supportive MHealth app for women in substance use recovery. *American Journal of Health Promotion*, *37*(1), 56–64. https://doi.org/10.1177/08901171221113834
26. Economou, D., Dwek, M., Roberston, C., Elliott, B., Kounenis, T., Azimi, T., Ramezanian, M., & Bell, N. (2017). PhytoCloud: A Gamified Mobile Web Application to Modulate Diet and Physical Activity of Women with Breast Cancer. In *2017 IEEE 30th International Symposium on Computer-Based Medical Systems(CBMS)*. https://doi.org/10.1109/cbms.2017.164
27. Elwahsh, S., Stern, N., Singh, A., & Ayobi, A. (2025). Linguistic Diversity and Mental Well-Being: Co-Designing Custom AI Chatbots with Multilingual Mothers. In *CUI ’25: Proceedings of the 7th ACM Conference on Conversational User Interfaces* (pp. 1–17). https://doi.org/10.1145/3719160.3736615
28. Fealy, S., Chan, S., Wynne, O., Dowse, E., Ebert, L., Ho, R., Zhang, M. W. B., & Jones, D. (2019). The Support for New Mums Project: A protocol for a pilot randomized controlled trial designed to test a postnatal psychoeducation smartphone application. *Journal of Advanced Nursing*, *75*(6), 1347–1359. https://doi.org/10.1111/jan.13971
29. Fietta, V., Rizzi, S., De Luca, C., Gios, L., Pavesi, M. C., Gabrielli, S., Monaro, M., & Forti, S. (2024). A chat-bot based version of the WHO-validated intervention Self-Help+ for stress management: Co-Design and Usability Testing (Preprint). *JMIR Human Factors*. https://doi.org/10.2196/64614
30. Figueroa, C. A., Luo, T. C., Jacobo, A., Munoz, A., Manuel, M., Chan, D., Canny, J., & Aguilera, A. (2021). Conversational Physical activity coaches for Spanish and English speaking women: a user design study. *Frontiers in Digital Health*, *3*. https://doi.org/10.3389/fdgth.2021.747153
31. Franco, P., Olhaberry, M., Kelders, S., & Muzard, A. (2023). A Chilean survey of perinatal women and health care professionals’ views towards perinatal apps. *mHealth*, *9*, 7. https://doi.org/10.21037/mhealth-22-37
32. Franco, P., Olhaberry, M., Kelders, S., Muzard, A., & Cuijpers, P. (2024). Guided web app intervention for reducing symptoms of depression in postpartum women: Results of a feasibility randomized controlled trial. *Internet Interventions*, *36*, 100744. https://doi.org/10.1016/j.invent.2024.100744
33. Franco, P., Olhaberry, M., Muzard, A., Harismendy, Á., & Kelders, S. (2024). Developing a guided web app for postpartum depression symptoms: User-Centered Design Approach (PREPrint). *JMIR Formative Research*, *8*, e56319. https://doi.org/10.2196/56319
34. Gewali, A., Lopez, A., Dachelet, K., Healy, E., Jean-Baptiste, M., Harridan, H., Evans, Y., Unger, J. A., Bhat, A., Tandon, D., & Ronen, K. (2021). A Social Media Group Cognitive Behavioral Therapy Intervention to Prevent Depression in Perinatal Youth: Stakeholder Interviews and Intervention Design. *JMIR Mental Health*, *8*(9), e26188. https://doi.org/10.2196/26188
35. Gilbert, S., Irvine, R., D’or, M., Adam, M. T. P., Collins, C. E., Marriott, R., Rollo, M., Walker, R., & Rae, K. (2023). Indigenous women and their nutrition during Pregnancy (The Mums and Bubs Deadly Diets Project): Protocol for a co-designed MHealth Resource Development study. *JMIR Research Protocols*, *12*, e45983. https://doi.org/10.2196/45983
36. Gilbert, S., Irvine, R., D’Or, M., Rae, K., & Murphy, N. (2023). Working with Indigenous Australian communities designing a nutritional mHealth tool during the Covid-19 pandemic. *AlterNative an International Journal of Indigenous Peoples*, *19*(3), 574–583. https://doi.org/10.1177/11771801231189346
37. Gill, R. K., Ogilvie, G., Norman, W. V., Fitzsimmons, B., Maher, C., & Renner, R. (2019). Feasibility and acceptability of a mobile technology intervention to support postabortion care (The FACTS Study Phase II) after surgical Abortion: User-Centered Design. *JMIR Human Factors*, *6*(4), e14558. https://doi.org/10.2196/14558
38. Gill, R., Ogilvie, G., Norman, W. V., Fitzsimmons, B., Maher, C., & Renner, R. (2019). Feasibility and Acceptability of a mobile technology intervention to support postabortion care in British Columbia: Phase I. *Journal of Medical Internet Research*, *21*(5), e13387. https://doi.org/10.2196/13387
39. Gill, R., Ogilvie, G., Norman, W. V., Fitzsimmons, B., Maher, C., & Renner, R. (2023). The FACTS Study Phase III: Feasibility and acceptability of myPostCare.ca – a comprehensive web-based intervention to support post-abortion care (Preprint). *JMIR Formative Research*. https://doi.org/10.2196/46284
40. Gordon, M., Henderson, R., Holmes, J. H., Wolters, M. K., & Bennett, I. M. (2015). Participatory design of ehealth solutions for women from vulnerable populations with perinatal depression. *Journal of the American Medical Informatics Association*, *23*(1), 105–109. https://doi.org/10.1093/jamia/ocv109
41. Horowitz, J., Posmontier, B., Geller, P., McDonough, M., Elgohail, M., Alvares, K., Wang, Y., Chang, K., & Ma, T. (2025). Translation of Mother-Baby Interaction Therapy-Interpersonal Psychotherapy (MBIPT) for Postpartum Depression from In-Person to Digital DeliveryMother-Baby Interaction Therapy via MommaConnect: Strategies to Translate a Face-to-Face Intervention to a Digital Healthcare Platform. *Medical Research Archives*, *14*(2). https://doi.org/10.18103/mra.v14i2.7298
42. Hossain, M. E., Najib, A. U., & Islam, M. Z. (2020). Combating domestic violence during COVID-19 pandemic in Bangladesh: Using a mobile application integrated with an effective solution. *2020 23rd International Conference on Computer and Information Technology(ICCIT)*, 1–6. https://doi.org/10.1109/iccit51783.2020.9392691
43. Houghton, L. C., Gibbons, M. P., Shekelle, J., Oakley-Girvan, I., Watterson, J. L., Magsamen-Conrad, K., Jones, C., & Gokal, K. (2021). Free Time For Wellness: a co-designed intervention utilizing social networks to encourage physical activity for cancer prevention among low resourced mothers. *BMC Public Health*, *21*(1). https://doi.org/10.1186/s12889-021-11775-9
44. Isaacs, K. R., Bajracharya, E., Taylor, S., Chang, K., Washio, Y., Parker, T., Paul, D. A., & X, T., MA. (2023). Usability and acceptability testing of a Plan of Safe Care in a mobile health platform. *Frontiers in Psychiatry*, *14*. https://doi.org/10.3389/fpsyt.2023.1182630
45. Jiang, L., Xu, J., Wu, Y., Liu, Y., Wang, X., & Hu, Y. (2024). Effects of the “AI-TA” Mobile App With Intelligent Design on Psychological and Related Symptoms of Young Survivors of Breast Cancer: Randomized Controlled Trial. *JMIR Mhealth and Uhealth*, *12*, e50783. https://doi.org/10.2196/50783
46. Lackie, M. E., Parrilla, J. S., Lavery, B. M., Kennedy, A. L., Ryan, D., Shulman, B., & Brotto, L. A. (2020). Digital Health needs of women with Postpartum Depression: focus group study. *Journal of Medical Internet Research*, *23*(1), e18934. https://doi.org/10.2196/18934
47. Leerlooijer, J. N., Kok, G., Weyusya, J., Bos, A. E. R., Ruiter, R. a. C., Rijsdijk, L. E., Nshakira, N., & Bartholomew, L. K. (2014). Applying Intervention Mapping to develop a community-based intervention aimed at improved psychological and social well-being of unmarried teenage mothers in Uganda. *Health Education Research*, *29*(4), 598–610. https://doi.org/10.1093/her/cyu020
48. Leger, L. D., Tryphonopoulos, P., Mackay, L., Beck, A., Moshipour, M., Poelzer, K., Dickey, A. P., Shillington, J., Linder, J., Deane, A., Wu, K., & Letourneau, N. (2022). User-Engaged design in the adaptation of VID-KIDS Home Visiting program for virtual delivery in the context of COVID-19. *The International Journal of Technology Knowledge and Society*, *18*(1), 49–73. https://doi.org/10.18848/1832-3669/cgp/v18i01/49-73
49. Liu, W., Huang, J., Pan, W., Zhu, Y., Li, M., & Xu, H. (2020). BRAVO: a physiological indication system for female college students to manage depression. In *Advances in intelligent systems and computing* (pp. 122–127). https://doi.org/10.1007/978-3-030-55307-4_19
50. Lyon, A. R., Munson, S. A., Renn, B. N., Atkins, D. C., Pullmann, M. D., Friedman, E., & Areán, P. A. (2019). Use of Human-Centered Design to Improve Implementation of Evidence-Based Psychotherapies in Low-Resource Communities: Protocol for Studies Applying a Framework to Assess Usability. *JMIR Research Protocols*, *8*(10), e14990. https://doi.org/10.2196/14990
51. M, M. S., Shyam, S., D, J. G., R, S., Jiwanti, P. K., & Raglend, J., I. (2025). Mind Ease: An Empathetic AI Companion for Women’s Mental Wellness. In *2025 Innovations in Power and Advanced Computing Technologies (i-PACT)* (pp. 1–6). https://doi.org/10.1109/i-pact65952.2025.11307990
52. Mahajan, M., Reddy, K., & Rajput, M. (2016). Design and implementation of a rescue system for safety of women. *2016 International Conference on Wireless Communications, SignalProcessing and Networking (WiSPNET)*, 1955–1959. https://doi.org/10.1109/wispnet.2016.7566484
53. Mancinelli, E., Gabrielli, S., & Salcuni, S. (2024). A Digital Behavioral Activation Intervention (JUNEX) for Pregnant women with subclinical depression symptoms: Explorative Co-Design Study. *JMIR Human Factors*, *11*, e50098. https://doi.org/10.2196/50098
54. Mancinelli, E., Magnolini, S., Gabrielli, S., & Salcuni, S. (2024a). A Chatbot (Juno) prototype to deploy a behavioral activation intervention to pregnant women: Qualitative evaluation using a multiple case study. *JMIR Formative Research*, *8*, e58653. https://doi.org/10.2196/58653
55. Mancinelli, E., Magnolini, S., Gabrielli, S., & Salcuni, S. (2024b). A Chatbot (Juno) prototype to deploy a behavioral activation intervention to pregnant women: Qualitative evaluation using a multiple case study. *JMIR Formative Research*, *8*, e58653. https://doi.org/10.2196/58653
56. Materia, F. T., Smyth, J. M., Heron, K. E., Hillemeier, M., Feinberg, M. E., Fonzi, P., & Downs, D. S. (2018). Preconceptional health behavior change in women with overweight and obesity: prototype for SMART strong healthy women intervention. *mHealth*, *4*, 24. https://doi.org/10.21037/mhealth.2018.06.06
57. McAlister, K., Baez, L., Huberty, J., & Kerppola, M. (2025). Chatbot to Support the Mental Health Needs of Pregnant and Postpartum Women (Moment for Parents): Design and Pilot Study. *JMIR Formative Research*, *9*, e72469. https://doi.org/10.2196/72469
58. McCall, T., Ali, M. O., Yu, F., Fontelo, P., & Khairat, S. (2021). Development of a mobile app to support self-management of anxiety and depression in African American women: Usability study. *JMIR Formative Research*, *5*(8), e24393. https://doi.org/10.2196/24393
59. McKellar, L., Steen, M., Charlick, S., Andrew, J., Altieri, B., & Gwilt, I. (2023). Yourtime: The development and pilot of a perinatal mental wellbeing digital tool using a co-design approach. *Applied Nursing Research*, *73*, 151714. https://doi.org/10.1016/j.apnr.2023.151714
60. Mckellar, L., Steen, M., & N, L. (2017). Capture my mood: a feasibility study to develop a visual scale for women to self-monitor their mental. . . *ResearchGate*. https://www.researchgate.net/publication/318055665_Capture_my_mood_a_feasibility_study_to_develop_a_visual_scale_for_women_to_self-monitor_their_mental_wellbeing_following_birth
61. Menon, B. M., Nampoothiri, A. K., F, F. L. K., B, G. D., & Rao, B. (2024). User-Centered Design of a Seasonal Calendar App for Women: Insights from a Case Study in India. *2024 IEEE Global Humanitarian Technology Conference (GHTC)*, 319–325. https://doi.org/10.1109/ghtc62424.2024.10771573
62. Moshki, M., Beydokhti, T. B., & Cheravi, K. (2013). The effect of educational intervention on prevention of postpartum depression: an application of health locus of control. *Journal of Clinical Nursing*, *23*(15–16), 2256–2263. https://doi.org/10.1111/jocn.12505
63. Mumbaikar, P., Yadav, T., Mugalikar, J., Sukale, A., & Lohiya, S. (2025). Women’s Wellness Guide Using Machine Learning. In *2025 International Conference on Emerging Trends in Industry 4.0 Technologies (ICETI4T)* (pp. 1–6). https://doi.org/10.1109/iceti4t63625.2025.11132227
64. Mustafina, S. N., Islam, M. N., Mahjabin, M. R., Mannan, M. M. R., & Islam, M. M. (2025). Identifying key indicators to develop a novel mobile application for early screening of postpartum depression in developing countries. *BMC Health Services Research*, *25*(1), 287. https://doi.org/10.1186/s12913-025-12429-x
65. Nicoletti, A. E., Rizzi, S., Fait, S., & Mayora-Ibarra, O. (2025). Prevention of Postpartum Depression via a Digital ACT-Based Intervention: Evaluation of a Prototype Using Multiple Case Studies. *Behavioral Sciences*, *15*(12), 1723. https://doi.org/10.3390/bs15121723
66. Nicoletti, A. E., Tonelli, M., Purin, B., & Rizzi, S. (2026). Digital Acceptance and Commitment Therapy for Lifestyle Change in Overweight Pregnant Women: A Feasibility Pilot Study. *Behavioral Sciences*, *16*(4), 585. https://doi.org/10.3390/bs16040585
67. Nuseibeh, B. Z., Johns, S. A., Shih, P. C., Lewis, G. F., Gowan, T. M., & Jordan, E. J. (2024). Co-Designing the MOSAIC mHealth App With Breast Cancer Survivors: User-Centered Design Approach. *JMIR Formative Research*, *8*, e59426. https://doi.org/10.2196/59426
68. Osman, A., Noor, N. M., Wahab, N. A., & Noraimi, N. I. A. (2023). Helping women towards healthy management of menopause: Design and development of mobile app for Menopausal Symptoms Tracker. In *2023 International Conference on University Teaching and Learning (InCULT)* (pp. 1–6). https://doi.org/10.1109/incult59088.2023.10482757
69. Osweiler, B. W., Rammaha, T., Szlyk, H. S., Dell, N. A., Macon, J., Jacobson, N. C., Burley, C., Goodman, M., Cavazos-Rehg, P. A., & Ramsey, A. T. (2025). Co-designing a mobile application to reduce self-stigma for people with opioid use disorder during pregnancy and the postpartum period. *Frontiers in Psychiatry*, *16*, 1607652. https://doi.org/10.3389/fpsyt.2025.1607652
70. Perego, P., & Terraroli, M. (2023). Integrated Design method and usability Outcomes of a mobile Application for Psychological Well-Being during Pregnancy. In *Wireless Mobile Communication and Healthcare* (pp. 287–303). https://doi.org/10.1007/978-3-031-32029-3_25
71. Pereira, J., Ambolkar, V. D., Bijja, A. J., & Bhatankar, T. R. (2026). Paripakvata: A Context-Aware Internet of Everything and Artificial Intelligence Integrated Solution for Holistic Pregnancy Care. In *2026 International Conference on Communication, Computing and Emerging Technologies (IC3ET)* (pp. 111–116). https://doi.org/10.1109/ic3et64989.2026.11467388
72. Perkes, S. J., Huntriss, B., Skinner, N., Leece, B., Dobson, R., Mattes, J., Hall, K., & Bonevski, B. (2022). Development of a Maternal and Child MHealth Intervention with Aboriginal and Torres Strait Islander Mothers: Co-design approach. *JMIR Formative Research*, *6*(7), e33541. https://doi.org/10.2196/33541
73. Perkes, S. J., Huntriss, B., Skinner, N., Leece, B., Dobson, R., Mattes, J., Hall, K., & Bonevski, B. (2023). Evaluation of an MHealth intervention (Growin’ Up Healthy Jarjums) designed with and for Aboriginal and Torres Strait Islander Mothers: Engagement and Acceptability Study. *JMIR Pediatrics and Parenting*, *6*, e43673. https://doi.org/10.2196/43673
74. Posmontier, B., Horowitz, J. A., Geller, P. A., Elgohail, M., McDonough, M., Alvares, K., Smoot, J., Chang, K., & Ma, T. (2024). Applying the PRECEDE-PROCEED model to develop MommaConnect: a digital healthcare platform for addressing postpartum depression and improving infant well-being. *Exploration of Neuroscience*, *3*(4), 309–320. https://doi.org/10.37349/en.2024.00052
75. Pourshahrokhi, N., Hunter, M., Farokhzadian, J., & Ahmadian, L. (2025). Identifying the content, capabilities, and design features of a mobile-based cognitive behavioral therapy intervention for managing menopausal symptoms. *International Journal of Medical Informatics*, *206*, 106163. https://doi.org/10.1016/j.ijmedinf.2025.106163
76. Ramachandran, R., Kunhardt, H., Chaltikyan, G., & Ivanova, O. (2026). Quality assessment of menopause apps using Mobile App Rating Scale (MARS) in India. *BMC Digital Health*, *4*(1). https://doi.org/10.1186/s44247-026-00246-z
77. Rau, N. M., Hasan, K., Ahamed, S. I., Asan, O., Flynn, K. E., & Basir, M. A. (2020). Designing a tablet-based prematurity education app for parents hospitalized for preterm birth. *International Journal of Medical Informatics*, *141*, 104200. https://doi.org/10.1016/j.ijmedinf.2020.104200
78. Reen, J. K., Chan, G., & Orji, R. (2024). iCare: Insights from the Evaluation of an App for Managing Stress Among Working-Class Indian Women. *International Journal of Human-Computer Interaction*, *41*(9), 5797–5816. https://doi.org/10.1080/10447318.2024.2366016
79. Reen, J. K., & Orji, R. (2022). Improving mental health among Working-Class Indian Women: insight from an interview study. *CHI Conference on Human Factors in Computing Systems Extended Abstracts*, 1–6. https://doi.org/10.1145/3491101.3519781
80. Rizzi, S., Fietta, V., Gios, L., Poggianella, S., Pavesi, M. C., De Luca, C., Marroni, D., Paoli, C., Gianatti, A., Burlon, B., Chiodega, V., Endrizzi, B., Giordano, A., Biagioli, F., Albertini, V., Purgato, M., Barbui, C., Guella, C., Gadotti, E., . . . Taddei, F. (2025). A Chatbot-Based version of a World Health Organization–Validated intervention (Self-Help Plus) for stress management in pregnant women: Protocol for a Usability study. *JMIR Research Protocols*, *14*, e53891. https://doi.org/10.2196/53891
81. Rizzi, S., Pavesi, M. C., Moser, A., Paolazzi, F., Marchesoni, M., Poggianella, S., Gadotti, E., & Forti, S. (2024). A Mindfulness-Based app intervention for Pregnant women: study design, development, and preliminary testing of a prototype (Preprint). *JMIR Formative Research*. https://doi.org/10.2196/58265
82. Rizzi, S., Poggianella, S., Pavesi, M. C., Gios, L., Bincoletto, G., Scolari, I., Paoli, C., Marroni, D., Tassinari, I., Baietti, B., Gianatti, A., Albertini, V., Burlon, B., Chiodega, V., Endrizzi, B., Benini, E., Guella, C., Gadotti, E., Forti, S., & Taddei, F. (2024). A Mindfulness-Based App Intervention for Pregnant Women: Protocol for a pilot feasibility study. *JMIR Research Protocols*, *13*, e53890. https://doi.org/10.2196/53890
83. Rodriguez, N. L., & Jareda, M. E. M. (2019). Design Process of an Alternate Reality Game (ARG) as a Strategy to Foster Social Support and Well-being of Mothers of Children with ASD. In *2019 IEEE 7th International Conference on Serious Games and Applications for Health (SeGAH)* (Vol. 5, pp. 1–7). https://doi.org/10.1109/segah.2019.8882440
84. Ronen, K., Gewali, A., Dachelet, K., White, E., Jean-Baptiste, M., Evans, Y. N., Unger, J. A., Tandon, S. D., & Bhat, A. (2023). Acceptability and utility of a digital group intervention to prevent perinatal depression in youths via Interactive Maternal Group for Information and Emotional Support (IMAGINE): pilot cohort study. *JMIR Formative Research*, *8*, e51066. https://doi.org/10.2196/51066
85. Ruben, J., Devadass, D., Goud, B. R., Navarro-Aguirre, Y., Kalidindi, B., Waghmare, A., Raj, T., Srinivasan, K., Collins, P. Y., & Bhat, A. (2025). Development of a Multiuser Interactive Health Response Application (MITHRA) for depression in women from a community-based organisation in India. *BJPsych Open*, *11*(2), e58. https://doi.org/10.1192/bjo.2025.8
86. Sarandos, S., Van Der Merwe, L. A., & Daniels, J. (2026). “I promise your secret is safe to me”: Understanding the intersections of disclosing HIV status, associated violence, and loved one’s support as a transgender woman in rural South Africa. *International Journal of Transgender Health*, 1–15. https://doi.org/10.1080/26895269.2026.2615647
87. Saravanan, K. A., Sathyasri, B., Mary, G. a. A., Farithkhan, A., Prasanna, N. V., & Ezilarasan, M. R. (2022). Women safety maneuver in real time scenarios. *2022 8th International Conference on Smart Structures and Systems (ICSSS)*, 1–5. https://doi.org/10.1109/icsss54381.2022.9782207
88. Savithri, C., S, L. B., Poornesh, B., & Velappan, V. (2023). Hope – An Arduino Based Safety device. *2023 Intelligent Computing and Control for Engineering and BusinessSystems (ICCEBS)*, 1–5. https://doi.org/10.1109/iccebs58601.2023.10448656
89. Scott, J., Oxlad, M., Dodd, J., Szabo, C., Deussen, A., & Turnbull, D. (2020). Creating Healthy Change in the Preconception Period for Women with Overweight or Obesity: A Qualitative Study Using the Information–Motivation–Behavioural Skills Model. *Journal of Clinical Medicine*, *9*(10), 3351. https://doi.org/10.3390/jcm9103351
90. Scott, J., Oxlad, M., Dodd, J., Szabo, C., Deussen, A., & Turnbull, D. (2022). Promoting health behaviour change in the preconception period: A combined approach to intervention planning (Preprint). *JMIR Formative Research*, *6*(4), e35108. https://doi.org/10.2196/35108
91. Senette, C., Buzzi, M. C., & Paratore, M. T. (2023). Self-assess momentary mood in mobile devices: a case study with mature female participants. *2023 3rd International Conference on Intelligent Communication andComputational Techniques (ICCT)*, 1–7. https://doi.org/10.1109/icct56969.2023.10075891
92. Shifflett, A., Karpisek, L., Patel, K., Cui, Y., Lawental, M., Wernette, G. T., Chang, H. K., Isaacs, K. R., & X, T., MA. (2025). Design of a mobile application for universal screening for women of child-bearing age engaged in comprehensive addiction and recovery environments (WE-CARE) for substance use and women from the general population. *Digital Health*, *11*, 20552076251318054. https://doi.org/10.1177/20552076251318054
93. Siddhpuria, S., Breau, G., Lackie, M. E., Lavery, B. M., Ryan, D., Shulman, B., Kennedy, A. L., & Brotto, L. A. (2022). Women’s Preferences and Design Recommendations for a Postpartum Depression Psychoeducation Intervention: User Involvement study. *JMIR Formative Research*, *6*(6), e33411. https://doi.org/10.2196/33411
94. Singleton, A. C., Raeside, R., Partridge, S. R., Hyun, K. K., Tat-Ko, J., Sum, S. C. M., Hayes, M., Chow, C. K., Thiagalingam, A., Maka, K., Sherman, K. A., Elder, E., & Redfern, J. (2022). Supporting women’s health outcomes after breast cancer treatment comparing a text message intervention to usual care: the EMPOWER-SMS randomised clinical trial. *Journal of Cancer Survivorship*, *17*(6), 1533–1545. https://doi.org/10.1007/s11764-022-01209-9
95. Singleton, A., Raeside, R., Partridge, S. R., Hayes, M., Maka, K., Hyun, K. K., Thiagalingam, A., Chow, C. K., Sherman, K. A., Elder, E., & Redfern, J. (2021). Co-designing a Lifestyle-Focused text message intervention for women after breast cancer Treatment: Mixed Methods study. *Journal of Medical Internet Research*, *23*(6), e27076. https://doi.org/10.2196/27076
96. Sreeraj, S. S., Unnikrishnan, A., Vishnu, K., Kennith, N. E., Anand, S., & Ramesh, M. V. (2020). Empowerment of Women Self Help Groups: Human Centered Design of a Participatory IoT solution. *2020 IEEE Global Humanitarian Technology Conference*, 1–8. https://doi.org/10.1109/ghtc46280.2020.9342858
97. Sugarman, D. E., Levine, E. A., Wang, C. L., Korte, F. M., Barbaro, N. A., Weiss, R. D., Marsch, L. A., Campbell, A. N., & Greenfield, S. F. (2026). Development of the Healthy Women Intervention to Increase Women’s Engagement in Medication Treatment for Opioid Use Disorder: Mixed Methods, User-Centered Design Approach. *JMIR Formative Research*, *10*, e85195. https://doi.org/10.2196/85195
98. Sugarman, D. E., Meyer, L. E., Reilly, M. E., & Greenfield, S. F. (2019). Feasibility and Acceptability of a Web-Based, Gender-Specific Intervention for Women with Substance Use Disorders. *Journal of Women S Health*, *29*(5), 636–646. https://doi.org/10.1089/jwh.2018.7519
99. Sugarman, D. E., Meyer, L. E., Reilly, M. E., Rauch, S. L., & Greenfield, S. F. (2021). Exploring Technology-Based Enhancements to Inpatient and Residential Treatment for Young Adult Women with Co-Occurring Substance Use. *Journal of Dual Diagnosis*, *17*(3), 236–247. https://doi.org/10.1080/15504263.2021.1940412
100. Sun, Y., Li, Y., Wang, J., Chen, Q., Bazzano, A. N., & Cao, F. (2021). Effectiveness of Smartphone-Based Mindfulness Training on Maternal Perinatal Depression: Randomized controlled trial. *Journal of Medical Internet Research*, *23*(1), e23410. https://doi.org/10.2196/23410
101. Tamanna, T. A., Choudhury, S., Afsana, N., & Khan, M. M. (2021). Mobile application based teli-nutrition system for COviD-19 pandemic. *2021 IEEE 12th Annual Information Technology, Electronics and Mobile Communication Conference (IEMCON)*, 0350–0356. https://doi.org/10.1109/iemcon53756.2021.9623177
102. Vikruthi, S., Suneetha, S., Basha, P. H., Sreelekha, B., Bruhati, B., & Asmitha, M. (2023). Design and development of IoT based smart Women Security Gadget. *2023 International Conference on Sustainable Communication Networks andApplication (ICSCNA)*, 1747–1753. https://doi.org/10.1109/icscna58489.2023.10370638
103. Zaman, K. T., Hasan, W. U., Bazlul, L., Motahar, T., & Ahmed, N. (2019). Exploring challenges and solution approaches regarding wellbeing of female Rohingya community in Bangladesh. *TENCON 2021 - 2021 IEEE Region 10 Conference (TENCON)*, 361–366. https://doi.org/10.1109/tencon.2019.8929413
104. Zimmermann, M., Yonkers, K. A., Tulu, B., Ford, L., Peacock-Chambers, E., Clare, C. A., Boudreaux, E. D., Lemon, S. C., & Byatt, N. (2025). Adapting an Anxiety Sensitivity Intervention for Perinatal Mental Health. *The Journal of Nervous and Mental Disease*, *213*(7), 167–179. https://doi.org/10.1097/nmd.0000000000001841
