# Supplementary material for: Designing for all: A scoping review and critical analysis on how digital mental health interventions serve (or fail) diverse women
Source: Internet Interv. 2026 Jul 16;45:100981. doi: 10.1016/j.invent.2026.100981 (PMC13448905; doi:10.1016/j.invent.2026.100981)
Supplement: Supplementary file 4 [file mmc4.docx]

**Supplementary Table 3: Coding of user involvement activities of the studies**

| **Study Title** | **Study Identifier** | **User involvement in the 'Integrate user insights phase'** | **User involvement in the 'Design phase' (e.g., co-creation sessions)** | **User involvement in the 'Assess phase' (e.g., usability tests, RCTs, user evaluation)** |
| --- | --- | --- | --- | --- |
| Women Safety Maneuver in Real Time Scenarios | Saravanan 2022 | No indication | No | no indication |
| Design and implementation of a rescue system for safety of women | Mahajan 2016 | No indication | no indication | no indication |
| Combating Domestic Violence during COVID-19 Pandemic in Bangladesh: Usinga Mobile Application integrated with an Effective Solution | Hossain 2020 | No indication | no | no |
| Hope: An Arduino Based Safety device | Savithri 2023 | No indication | No | No |
| PhytoCloud: A Gamified Mobile Web Application to Modulate Diet andPhysical Activity of Women with Breast Cancer | Economou 2017 | No indication; conducted **focus groups with multi-disciplinary stakeholders (no user involvement)** | no indication | Planned. User testing trials will be conducted with self-help cancer groups. |
| Design and Development of IoT based Smart Women Security Gadget | Vikruthi 2023 | No indication | no indication | no indication |
| Mobile Application Based Teli-nutrition System for Covid-19 Pandemic | Tamanna 2021 | No indication | no | no |
| Empowering Safety: Arduino-Based Alert System Design and Implementationfor Women | Chandra 2023 | No indication | no indication | no indication |
| Self-assess Momentary Mood in Mobile Devices: a Case Study with MatureFemale Participants | Senette 2023 | no indication | when designing the app, there are two interactive widgets of the app were refined through the **questionnaire** with women aged 45-65 | **Usability/User experience test.** The study analyzed the participants' in-app input (mood recorded) and their subjective evaluation of the usability/user experience (likert scale questionnaire). There is no mention of specifically considering a diverse target groups, besides focusing on older females |
| Helping Women Towards Healthy Management of Menopause: Design andDevelopment of Mobile App for Menopausal Symptoms Tracker | Osman 2023 | no indication | no indication | no indication |
| Exploring Challenges and Solution Approaches Regarding Wellbeing of FemaleRohingya Community in Bangladesh | Zaman 2019 | Rohingya women are involved in the **survey study** (N=117)and **semi-structured interviews** (N=10) | no indication | no indication |
| Supporting women's health outcomes after breast cancer treatment comparinga text message intervention to usual care: the EMPOWER-SMS randomisedclinical trial | Singleton 2023 | no indication | **co-design workshop** with end-users, health professionals, and researchers to draft text messages, and then the messages are edited based on users evaluation outcomes | Yes, Single-blind **RCT** (N=160). |
| Development of an Electronic Screening and Brief Intervention to AddressPerinatal Substance Use in Home Visiting: Qualitative User-CenteredApproach | Dauber 2022 | yes, through **semi-structured Interviews** in the 1st cycle to re-design an existing system and develop their own intervention. | yes, through **semi-structured Interviews** in the 2nd iterative design cycles to refine the prototype. Not specifically diverse population | yes, **pilot test** in New Jersey with 20 home visitors and 10 clients |
| Usability and acceptability testing of a Plan of Safe Care in a mobilehealth platform | Isaacs 2023 | no indication. Mentioned that the previous study conducted UCD to define design features | no indication. Mentioned that the previous study conducted UCD to define design features | Yes, **usability and acceptability tests** with end users |
| mHealth for maternal mental health: Everyday wisdom in ethical design | Barry 2017 | Yes. Through **17 Iterative Design sessions** were carried out with 8 pregnant women and 2 mothers, to explore design challenges of self-report of wellbeing | yes,Through **17 Iterative Design sessions** were carried out with 8 pregnant women and 2 mothers, users reviewed the low-fi prototype and provided feedback | **Feasibility Study** with 355 women |
| Applying Intervention Mapping to develop a community-based interventionaimed at improved psychological and social well-being of unmarried teenagemothers in Uganda | Leerlooijer 2014 | The previous need assessment qualitative study involved **focus groups** and **interviews** with female adolescents aged 15–19 years (N = 85), also with community member (N=12) | A **multi-displinary planning group** was established (including teenage mothers); The planning group designed the intervention and iteratively added new components between 2000 and 2011 | A qualitative **effect evaluation** was conducted in March 2012, between March 2000 and March 2012, 1036 unmarried teenage mothers participated |
| Participatory design of eHealth solutions for women from vulnerablepopulations with perinatal depression | Gordon 2016 | Yes, end-users are involved from the start of the design process as **members of the participatory group meetings throughout the process, with montly meetings**. Including collectively writing a mission statement, which included the objective of improving communication and healthy outcomes in women with emotional distress using easilyaccessible interactive technologyAnd they participated in monthly group meetings focused on generating ideas and providing feedback on brief pilot studies implemented by providers, clinical administrative staff, health information technology developers, and video producers. Meetings were facilitated by the research team and involved brainstorming, problem-solving, role-playing, and feedback on content. However demographics of the SPIRIT groups members are not specified. | Yes, end-users are involved in the **participatory group meetings**. Patient participants were critical to identifying favored language, features, and stylistic elements of the â€œlookâ€ of the app that were appropriate to the target population. Input on readability and literacy challenges with language used in the app were particularly targeted by these members of the group. | Yes, through the **participatory group meetings**, they participated in iterative evaluating and improving the prototypes. |
| Social interaction needs and entertainment approaches to pregnancywell-being in mhealth technology design for low-income transmigrant women:Qualitative codesign study | Aljaberi 2018 | **Focus groups** were used as a warm up for engagement in the second phase of codesign workshops. Participants were asked to discuss their prenatal experiences as recent immigrants related to topics of pregnancy, relationships, and technology. | **Co-design workshops**. The participants were divided into two groups with 2 participants each to ensure equal participation. Each group was asked to come up with design solutions reflecting themes discussed in phase 1 (prenatal experiences as recent migrants). Then, the groups were asked to exchange designs, critique them, and revise them if they were so inclined. | no indication |
| The effect of educational intervention on prevention of postpartumdepression: An application of health locus of control | Moshki 2014 | Yes, conducted **a pilot study and need assessment** of pregnant mothers, but vaguely reported | no indication | Yes, **RCT** |
| Free Time For Wellness: a co-designed intervention utilizing socialnetworks to encourage physical activity for cancer prevention among lowresourced mothers | Houghton 2021 | **semi-structured interviews** are conducted to identify women's barriers and facilitators of free time for physical activity | **co-design workshop** with women are conducted for intervention development | yes. **Feasibility test** with 21 mothers |
| Digital support for living with and beyond gynaecological cancer | Ashmore 2020 | Yes, **co-design workshops**. Users were invited to participate in workshop 2 (establishing key areas for supporting women following treatment for gynealogical cancer). | Yes, **co-design workshops**. Users were involved in workshop 3 (developing design brief and system requirements) and workshop 4 to review the intial app wireframes | No indication |
| The Support for New Mums Project: A protocol for a pilot randomized controlled trial designed to test a postnatal psychoeducation smartphoneapplication | Fealy 2019 | no indication | No indication. Mentioned App development with multidisciplinary experts and childbearing women, couldn't find earlier relevant publications. | No indication. Planned to do pilot RCT. Couldn't find later relevant publications |
| Development of a mobile app to support self-management of anxiety anddepression in African American Women: Usability study | McCall 2021 | **Need assessment through focus groups** with 20 users; a web survey of 395 women examining user needs towards the app requirement. | No indication. Prototype built by multidisplinary researchers | Yes, **usability test** with 15 users, through cognitive walkthrough, think-aloud, eye tracking and QUIS. Feedback used for refinement |
| Bravo: A physiological indication system for female college students tomanage depression | Liu 2021 | Yes, **questionnaires** (n=200) and 3 **focus groups** (n=7 each) were implemented to generate user insights. | no indication | **Prototype try-out** with 10 female college students |
| Conversational Physical Activity Coaches for Spanish and English SpeakingWomen: A User Design Study | Figueroa 2021 | Yes, **semi-structured interviews with 18 low-income women,** where women were asked about their opinions and knowledge of chatbots | Yes, The researchers developed an initial prototype. Then the users were involved in a **co-design workshop** where they added ideas for chatbot use and design; And **Usability test**, User were involved in the design activities including a Wizard-of-oz procedure where they tested the chatting behavior of the agent; also in **post-test semi-structure interviews** where they gave opinions of the chatbot prototype. | No indication. Planned future work involving RCT and larger user testing |
| A Counseling Mobile App to Reduce the Psychosocial Impact of HumanPapillomavirus Testing: Formative Research Using a User-Centered DesignApproach in a Low-Middle-Income Setting in Argentina | Antelo 2022 | **Yes, 29 semistructured interviews and 4 online Focus groups** about user needs and experiences surrounding HPV testing | Yes, **Focus group participatory evaluation of seven app mock-ups and refining** | No indication, no functional prototype/mvp exists yet |
| ISDT Case Study of We'll App for Postpartum Depression Women | Chiou 2021 | No indication. Though they specified the activities conducted to integrate user insights conducted by a "cross-sector team" (no indication of whom are involved). | No indication. They reported conducting "user-oriented activities", such as Value Proposition, prototyping, Value Opportunity Analysis etc, by a multidisciplinary design team, there is no indication of user involvement in this process. | no indication |
| Designing a tablet-based prematurity education app for parentshospitalized for preterm birth | Rau 2020 | Yes, through **5 focus group meetings** to assess the information needs of the target audience (not reported in this article but in a previous study). | Yes, a **focus group** made up of 5 parents of premature infants, 3 medical providers, and one software engineer was formed, to design information content and format of the app. After that, multiple **co-creation meetings** with users to iteratively develop and refine prototypes (low and high fidelity). | Yes, through **usability tests** across the course of development, 13 users tested the high-fidelity prototype through 2 rounds of testing, in which thinking aloud methodology was performed. |
| User-Engaged Design in the Adaptation of VIDKIDS Home Visiting Program forVirtual Delivery in the Context of COVID-19 | Duffett-Leger 2020 | Users (mothers) were involved in **focus group interviews** | Users were directly involved in the **13 prototyping sessions** | Yes, **usability test**. Users were directly involved in the usability testing and pilot testing of the platform |
| Empowerment of Women Self Help Groups: Human Centered Design of aParticipatory IoT solution | Sreeraj 2020 | No indication. Participatory requirements gathering with self-help group administrators and stakeholders to understand the organization being designed for, but no end-user involvement | No indication. Researcher conducted the design process | No indication, the application has yet to be implemented and tested |
| Digital health needs of women with postpartum depression: Focus groupstudy | Lackie 2021 | **focus groups** were conducted in 7 communities(n=31 women) across BC for need assessment and generating design requirements | yes, **focus groups with 9 women** to review and gather feedback on the webpage prototypes. | no indication |
| Effectiveness of smartphone-based mindfulness training on maternalperinatal depression: Randomized controlled trial | Sun 2021 | no indication | no indication, expert adapted the interventions, no end-user participation | Yes, through **RCT** to evaluate the intervention's effectiveness. |
| Co-designing a lifestyle-focused text message intervention for women afterbreast cancer treatment: Mixed methods study | Singleton 2021 | yes, through 1 **co-design workshop** (n=2 breast cancer survivors, plus 1 citizen collaborator, 4 health professionals, and researchers) to determine the SMS program structure | Yes, **co-design workshops** for the text message evaluation with 14 breast cancer survivors; And s**emi-structured interviews** for qualitative evaluation of the contents (n=3 consumers) | Yes, **RCT** with 160 women |
| Evaluation of an mHealth Intervention (Grow-Up Healthy Jarjums)Designed With and for Aboriginal and Torres Strait Islander Mothers:Engagement and Acceptability Study | Perkes 2023 | Yes, through **Focus groups** with 31 Aboriginal and Torres Strait Islander women and 11 health professionals across three communities using storyboards, card sorting, and participatory design activities to identify health information needs, preferred delivery modes, cultural preferences, and intervention requirements | Yes, through **Participatory co-design workshops** (31 women, 11 health professionals) to iteratively developed and refined the prototype | Yes, 4-week **pilot study** with 41 Aboriginal and Torres Strait Islander mothers, followed by **post-intervention semi-structured interviews** with 32 mothers evaluating engagement, usability, acceptability and cultural appropriateness |
| Applying User-Centered Design in the Development of a Supportive mHealthApp for Women in Substance Use Recovery | Eaves 2023 | Yes, 5 **focus groups** (n=27 women in recovery; 3–7 participants per group) generating initial design requirements. | Yes, focus groups for rapid iterative refinement. Same participants as before | Yes, **Usability test**. After initial focus groups and incorporation of feedback into a working prototype, conducted 4 additional beta testing groups of 3-5 women each. |
| A Web-Based Intervention to Address Risk Factors for Maternal Morbidityand Mortality (MAMA LOVE): Development and Evaluation Study | Amore 2023 | Yes, they test the concept for the tool running 5 **focus groups** with Black birthing parents as well as health care providers, midwives and community organisations | No indication. The 1st prototype was iterately designed and evaluated by mix of stakeholders (nurses, engineers, community experts...) but not the end user | No indication. |
| Yourtime: The development and pilot of a perinatal mental wellbeingdigital tool using a co-design approach | McKellar 2023 | **Feasibility testing** of the paper-based tool followed by one **co-design workshop** (n=4 mothers) generating app concepts and requirements | One co-design workshop (n=4 mothers) informing interface and feature design, followed by researcher/developer-led prototype development | **Pilot evaluation** of Version 1.0 with 14 end users assessing usability, acceptability, functionality, and perceived usefulness |
| Integrated Design Method and Usability Outcomes of a Mobile Applicationfor Psychological Well-Being During Pregnancy | Perego 2023 | yes, 3 **focus groups** (n=19 mothers; n=17 healthcare professionals) identifying requirements. | Yes, iterative evaluation of mockups and prototypes by the same stakeholder groups, supported by **interviews and questionnaires** | Yes, **usability test** with 30 women. |
| Adaptive Text Messaging for Postpartum Risky Drinking: Conceptual Modeland Protocol for an Ecological Momentary Assessment Study | Dauber 2022 | Yes, **survey** with 165 postpartum women on their drinking behaviours, mobile technology use and intervention preferences; And a 14-day **ecological momentary assessment (EMA)** with 30 postpartum women to understand contextual factors and refine intervention requirements | No indication. Planned participatory design with focus groups | No indication |
| Promoting Health Behavior Change in the Preconception Period: CombinedApproach to Intervention Planning | Scott 2022 | Yes, 23 qualitative **semi-structured interviews for** needs assessment; intervention objectives and logic model derived from user insights | No indication. Researchers and experts stakeholders conducted the iterative design process | Informal pilot testing of branding and interface concepts (n=9 women) |
| Indigenous Women and Their Nutrition During Pregnancy (the Mums and BubsDeadly Diets Project): Protocol for a Co-designed mHealth ResourceDevelopment Study | Gilbert 2023 | Yes. in Phase 1 **focus groups and social groups** are organized with the target group (N=48) "to develop a deep understanding of community identified needs and priorities around nutrition in pregnanc"" | No indication. Not yet proceed to phase 2, planned participatory co-design workshops using personas. | No indication. Not yet proceed to assess phase |
| Feasibility and Acceptability of a Mobile Technology Intervention to Support Postabortion Care After Surgical Abortion (the FACTS Study Phase 3): Mixed Methods Prospective Pilot Study | Gill 2024 | Yes, **Survey + semi-structured interviews** with target users (n=58) about needs and preferences | Yes, **iterative prototype usability testing (not MvP)** and feedback with 9 women | Yes, **Pilot evaluation** of myPostCare with surveys and interviews (n=93). |
| Mobile Mental Health in Women's Community-Based Organizations: Protocol for a Pilot Randomized Controlled Trial | Bhat 2023 | Yes, through **semi-structured interviews** together with **two focus groups** (69 women from 5 villages) to inform app requirements and content. | Participatory design **focus groups** (5-6 end users: SHG members and community health workers) to iteratively review app prototypes, resulting in refinement of the final app before pilot deployment | yes, through pilot RCT, 85 women |
| Improving Mental Health Among Working-Class Indian Women: Insight From An Interview Study | Reen 2022 | Yes, **pre-study questionnaire** (n=37) collected participant demographics and awareness of existing mental health apps; and **semi-structured interviews** (n=31 working-class Indian women) to generate user requirements and design implications. | Yes, **iterative prototype usability evaluation (not MvP)** (n=40) and informed the refinement of the prototype | Yes,through a two-week **field deployment**, 30 women used iCare in daily life for two weeks, followed by a **post-use questionnaire** and **follow-up interviews** (n=22) |
| A Digital Behavioral Activation Intervention (JuNEX) for Pregnant Women With Subclinical Depression Symptoms: Explorative Co-Design Study | Mancinelli 2024 | no indication. The intervention was primarily derived from literature and ORBIT framework | Yes, through **iterative prototype usability test** (11 pregnant women), not yet MvP | Yes, usability and user engagement tests; |
| Designing of user interface and user experience in prevent sexual violence application (cekal) using the design thinking method (case study: Region of Bandar Lampung) | Adrian 2024 | Yes, **Interviews** were conducted with victims of sexual violence (Sample not specified) | no indication. The design decisions are primarily made by researchers and informed by earlier interviews results | yes, two rounds of **usability tests** evaluating effectiveness, efficiency, and satisfaction (SUS) |
| Developing a Guided Web App for Postpartum Depression Symptoms: User-Centered Design Approach | Franco 2024 | Yes, **a web-based survey (n=451)** was conducted, followed by a Value Specification phase with 8 web based focus groups (n=31) | Yes, through **2 focus groups** with postpartum women, they reviewed illustrations, photographs, screenshots of the Workbook etc.; 5 iterative prototype usability test the high-fidelity clickable Figma prototype. | yes, **Feasibility RCT** (n=65) |
| A Chatbot-Based Version of the World Health Organization “Validated Self-Help Plus Intervention for Stress Management: Co-Design and Usability Testing | Fietta 2024 | Yes, **exploration** with user representatives, health care providers, and eHealth experts to gather needs and adaptation requirements for translating WHO SH+ into a chatbot-based intervention | Yes, target users (n=15) participated in the **iterative development and high-fidelity prototype test** | No indication, a usability study with 50 women is planned (from the protocol paper) |
| User-Centered Design of a Seasonal Calendar App for Women: Insights from a Case Study in India | Menon 2024 | no indication | no indication | yes, **exploration and survey**. participants explored the app's features and functions. Followed by a survey to assess participants' perceptions of the app |
| Preconceptional health behavior change in women with overweight and obesity: prototype for SMART strong healthy women intervention. | Materia 2018 | **Semi-structured focus group interviews** to identify users'preferred program aspects. Participants reviewed the existing Strong Healthy Women intervention and discussed preferences for receiving content through smartphones. | yes, **through the same focus groups interviews** as the Integrate phase; Women generated redesign suggestions and feature recommendations. Their feedback directly informed prototype development, and 87% of redesign preferences were incorporated. | no indication, but the authors explicitly state that development and testing are future steps. |
| A Mindfulness-Based App Intervention for Pregnant Women: Qualitative Evaluation of a Prototype Using Multiple Case Studies. | Rizzi 2025 | no indication, the intervention contents are developed by multi-displinary researchers | Yes, they evaluated the paper prototype and lo-fi mockups instead of MVP, through surveys, usage logs and interviews; Women provide extensive feedback but do not influence design decisions during the study. | no indication, they plan to conduct a proof-of-concept study with about 50 women in the future. |
| Chatbot to Support the Mental Health Needs of Pregnant and Postpartum Women (Moment for Parents): Design and Pilot Study. | McAlister 2025 | Yes. **Ethnographic interviews** (n=43) to explore participants’ mental health needs, challenges during pregnancy or post partum, and expectations for digital support tools. | Yes, through **iterative prototype testing**. A first prototype was developed and used by participants for 4 weeks. Then they iterated and a second version of the prototype was adopted after 4 weeks based on participant feedback. | yes, **pilot testing** with the functional prototype, women used the chatbot for a month then completed **post-use survey** for feedback collection |
| Align: Empowering High School Students with PMS Awareness and Holistic Support | Dharani 2025 | **Semi-structured interview (N=7 ), survey (N=15)** with women to gather personal experiences, societal attitudes, and medical perspectives. | **usaibility testing** with 10 users, no indication wether a MVP or prototype; user has very limited involvement by providing feedback on usability | no indication |
| Women’s Wellness Guide Using Machine Learning | Mumbaikar 2025 | no indication | no indication | no indication |
| Adapting an Anxiety Sensitivity Intervention for Perinatal Mental Health: Development of a Digital Intervention. | Zimmermann 2025 | yes, through **semi-structured interviews** with perinatal individuals experiencing economic marginalization and advisory council inputs | yes, through **advisory council meeting**, women provided Iterative prototype refinement; And they also conducted **user testing** (different group from the council), with pre-interview surveys, think-aloud user testing and interview and post-terview survey for acceptability, usability and personalization preferences. | No, a functional prototype haven't been developed yet. |
| Mind Ease: An Empathetic AI Companion for Women's Mental Wellness | Subashini 2025 | Yes, through 25 **semi-structured interviews** with women and 200 surveys for women's need assessment, claimed these data was triangulated with clinical literature to define initial design criteria | yes, limited, "selected participants" provided user feedback during the Agile development, which informs the iterative content refinement | yes, they did **pilot testing** with the beta-version (2-week closed beta test was conducted with 60 women participants). |
| GETO Application Development: A CulturallyGrounded Gamified Exposure Therapy Platform for Managing Anxiety in Functional Gastrointestinal Disorder | Ardi 2025 | Yes , a needs analysis was conducted involving 335 productive-age female respondents (a **clinical assessment survey**) from Indonesia, Nigeria, South Africa, and Turkey who experienced FGID and GAD. But it's measuring psychological conditions. | Yes, limited, "initial validation was conducted through a **user test** by a multidisciplinary team consisting of specialist physicians, counselors, and patient users." | yes, they did a **trial** with 335 productive-age female patients from four countries, using a quantitative pretest-posttest approach and Partial Least Square Structural Equation Modeling (PLSSEM) structural analysis. |
| Towards effective digital lifestyle interventions for pregnant women with obesity: A qualitative study exploring women's and healthcare providers' perspectives. | de Bruin 2026 | yes, through **focus groups** and **semi-structured interviews** with 13 women, exploring healthy lifestyle behaviours, barriers, lifestyle care experiences, app experiences, needs and preferences; Women's inputs shaped the app feature, personalization, contents, language, communication style, and support needs. | yes, through the **focus groups**, Participants reviewed screenshots/examples, categorized proposed features into essential/desirable/unnecessary, and suggested additional features. | no indication, not yet |
| Digital Acceptance and Commitment Therapy for Lifestyle Change in Overweight Pregnant Women: A Feasibility Pilot Study | Nicoletti 2026 | no indication, they developed the intervention based on literature and consulted multidisplinary experts | yes, 4 women **tested the prototype** (not mvp) and provided feedback; | no indication, Although the authors call this study a "feasibility pilot", this study is fundamentally an iterative prototype evaluation |
| Paripakvata: A Context-Aware Internet of Everything and Artificial Intelligence Integrated Solution for Holistic Pregnancy Care | Pereira 2026 | no indication | no indication | no indication |
| A Culturally Sensitive Interactive Digital Narrative to Promote Bodily Awareness Among Afghan Women | Anwar 2025 | yes, **semi-structured interview** with 5 Afghan women for investigating their needs, especially the culture- and context-related ones, such as phone surveillance, cultural stigma menstruation taboos, emotional suppression, privacy concerns | yes, **iterative prototype testing** throughout the design phases, women tested and provided feedback for Prototype 1, Prototype 2, Prototype 3, and Final prototype. Each prototype was iterated with user feedback afterwards. Using Scenario-based Think Aloud sessions to evaluate the final prototype. | no indication, not yet, no evidence of deployed MVP |
| Translating Combined Mother-Baby Interaction Therapy and Interpersonal Psychotherapy for Postpartum Depression from In-Person to Digital Delivery via MommaConnect. | Horowitz 2026 | yes, through **focus groups** and **Community Advisory Board (CAB) meetings** to understand women's treatment needs and barriers to care, | yes, through **Co-design workshops** and feedback cycles. Women reviewed: low-fidelity wireframes, content, app features, workflows, and interface designs | yes, **Usability evaluation of the functional prototype**. Participants reviewed prototypes and completed System Usability Scale assessments |
| Identifying the content, capabilities, and design features of a mobile-based cognitive behavioral therapy intervention for managing menopausal symptoms. | Pourshahrokhi 2026 | yes, 7 menopausal women were involved in **Semi-structured interviews** exploring: information needs, symptom management needs, desired app functions, preferred interaction styles, barriers, and preferences | no indication. No actual design artifacts were produced in this paper. | no indication. No Design phase yet. |
| Development of the Healthy Women Intervention to Increase Women's Engagement in Medication Treatment for Opioid Use Disorder: Mixed Methods, User-Centered Design Approach. | Sugarman 2026 | yes, 20 women with current OUD participated in **semi-structured interviews** for needs and barries assessment. And they described how interview themes informed the intervention modules and contents | no indication. | 2020 prepilot (n=30) 2020 pilot (n=60) 2026 beta testing (n=5) later pilot trial planned |
| Design Process of an Alternate Reality Game (ARG) as a Strategy to Foster Social Support and Well-being of Mothers of Children with ASD | Rodríguez 2019 | **In person focus group** (n=7 mothers) to explore worries and stresses of women; **Online WhatsApp focus group (n=11 mothers, 1 week)** discussed coping strategies, social support and desired course content etc. | **Participatory co-design workshops** using focus-group findings (n=11 mothers) directly informed game scope and features | no indication, not yet |
| A social media group cognitive behavioral therapy intervention to prevent depression in perinatal youth: Stakeholder interviews and intervention design | Gewali 2021 | 21 semistructured individual interviews with 10 pregnant or postpartum youths to provide design recommendations | no indication | **pilot cohort study** with 10 participants, they completed the 12-week intervention followed by **questionnaires** and semi-structured **interviews** |
| Effects of the “AI-TA” Mobile App With Intelligent Design on Psychological and Related Symptoms of Young Survivors of Breast Cancer: Randomized Controlled Trial | Jiang 2024 | Need assessment of young breast cancer survivors (User study in 2022) | no indication. The app was developed by the research team | RCT with user evaluation, satisfaction questionnaire, and outcome assessment (n=124) |
| Co-Designing the MOSAIC mHealth App With Breast Cancer Survivors: User-Centered Design Approach. | Nuseibeh 2024 | **They conducted 5 UCD sessions in total, end-users (5 breast cancer survivors) were consistently involved throughout. UCD session** 1: problem identification) | **UCD Session** 2-4: solution generation, convergence, and prototyping | **UCD session** 5: Debriefing and evaluation. one-week prototype use and complete the System Usability Scale (SUS); the Acceptability E-scale |
| Developing a Mood and Menstrual Tracking App for People With Premenstrual Dysphoric Disorder: User-Centered Design Study. | Apsey 2024 | **one-on-one semi-structured interviews (n=4 users)** exploring user experiences of menstrual mood tracking apps usage and preferences | A **co-design workshop** with 8 women, to identify preferred app features | no indication |
| Linguistic Diversity and Mental Well-Being: Co-Designing Custom AI Chatbots with Multilingual Mothers | Elwahsh 2025 | Individual co-design sessions with 6 participants. Session 1: understanding users' emotional and Linguistic Needs; Session 2: Storyboarding Lived Experiences and AI Integration Points | Co-design Session 3 (n=6): Co-Creating Custom AI Chatbot Prompts | Co-design Session 4 (n=6): Evaluating Custom AI Chatbots |
| Little Cloud: A Digital Companion App to Support Those Experiencing Pregnancy Loss | Wang 2025 | user research through p**ublic discussions and in-depth semi-structured interviews** with individuals (N=13) who have experienced miscarriage | Participants (N=11) interacted with the first prototype (developed by researchers) during 1.5-hour sessions and provided refinement suggestions | No indication. Mentioned 30-day extensive testing with professionals in physical and mental health |
| A Conceptual Design Framework of GorbhoShongi App for Mental Well-Being Among Bangladeshi Pregnant and Postpartum Women | Ahmed 2024 | no indication | no indication. The intervention was developed by the research team | no indication. planned to do longitudinal study |
| Tailoring a Skills-Based Serostatus Disclosure Intervention for Transgender Women in South Africa: Protocol for a Usability and Feasibility Study | Daniels 2025 | **Semi-structured qualitative interviews** with trans women living with HIV-positivestatus (n = 21) were conducted in South Africa. Questions addressed experiences of GBV/IPVand the influence of loved one’s support. | no indication nor planned to do. | No indication. Planned to conduct usability testing |
| Prevention of Postpartum Depression via a Digital ACT-Based Intervention: Evaluation of a Prototype Using Multiple Case Studies | Nicoletti 2025 | no indication | Women (n=7 mothers) and healthcare professionals (midwives) participated in **iterative formative evaluation** to refine chatbot content, accessibility, usability, language, and interaction. | No indication |
| Design of a mobile application for universal screening for women of child-bearing age engaged in comprehensive addiction and recovery environments (WE-CARE) for substance use and women from the general population. | Shifflett 2025 | **E-surveys (n = 467)** regarding women's communication behavior of their alcohol/substance use with their doctors | **Focus group 1 with women aged 18-44 (n = 30) to get users' insight** ón WE-CARE's design and features; Focus group 2 collected users feedback on low-fidelity wireframes | no indication |
| Co-designing a mobile application to reduce self-stigma for people with opioid use disorder during pregnancy and the postpartum period. | Osweiler 2025 | quantitative baseline questionnaires for need assessment | structured **questionnaire** to understand perceived ease of use, usefulness, and intervention acceptability; They also completed a **semi-structured interview** about which digital intervention components were valued and which needed adaptation | **pilot test** with a fully function application for outcome and acceptibility evaluation |
| Identifying key indicators to develop a novel mobile application for early screening of postpartum depression in developing countries. | Mustafina 2025 | **semi-structured interviews** with both doctors and PPD patients, to evaluate the credibility and relevance of the EPDS' statements in the context of detecting PPD in Bangladesh | no indication | **Evaluation study** with 45 new mothers of the postpartum period. Group sessions were held with 10–12 participants at a time to evaluate the system's performance. |
