# Supplementary material for: Designing for all: A scoping review and critical analysis on how digital mental health interventions serve (or fail) diverse women
Source: Internet Interv. 2026 Jul 16;45:100981. doi: 10.1016/j.invent.2026.100981 (PMC13448905; doi:10.1016/j.invent.2026.100981)
Supplement: Supplementary file 5 [file mmc5.docx]

**Overview of demographics data extracted from the included studies (N=77)**

| **Study Title** | **Study Identifier** | **Mean age** | **Racial/ethnic background** | **Immigration status** | **Annual income (or of their parents)** | **Education level** |
| --- | --- | --- | --- | --- | --- | --- |
| Women Safety Maneuver in Real Time Scenarios | Saravanan 2022 | no indication | no indication | no indication | no indication | no indication |
| Design and implementation of a rescue system for safety of women | Mahajan 2016 | no indication | no indication | no indication | no indication | no indication |
| Combating Domestic Violence during COVID-19 Pandemic in Bangladesh: Usinga Mobile Application integrated with an Effective Solution | Hossain 2020 | no indication | no indication | no indication | no indication | no indication |
| Hope â€“ An Arduino Based Safety device | Savithri 2023 | no indication | no indication | no indication | no indication | no indication |
| PhytoCloud: A Gamified Mobile Web Application to Modulate Diet andPhysical Activity of Women with Breast Cancer | Economou 2017 | no indication | no indication | no indication | no indication | no indication |
| Design and Development of IoT based Smart Women Security Gadget | Vikruthi 2023 | no indication | no indication | no indication | no indication | no indication |
| Mobile Application Based Teli-nutrition System for Covid-19 Pandemic | Tamanna 2021 | no indication | no indication | no indication | no indication | no indication |
| Empowering Safety: Arduino-Based Alert System Design and Implementationfor Women | Chandra 2023 | no indication | no indication | no indication | no indication | no indication |
| Self-assess Momentary Mood in Mobile Devices: a Case Study with MatureFemale Participants | Senette 2023 | mean = 55.67 | no indication | no indication | no indication | no indication |
| Helping Women Towards Healthy Management of Menopause: Design andDevelopment of Mobile App for Menopausal Symptoms Tracker | Osman 2023 | no indication | no indication | no indication | no indication | no indication |
| Exploring Challenges and Solution Approaches Regarding Wellbeing of FemaleRohingya Community in Bangladesh | Zaman 2019 | no indication | Rohingya | Refugees who currently lives in Bangladesh | no indication | no indication |
| Supporting women's health outcomes after breast cancer treatment comparinga text message intervention to usual care: the EMPOWER-SMS randomisedclinical trial | Singleton 2023 | mean = 54,8 | Caucasian (44%), South asian (13%), other asian (36%), other (20%) | Australian (41%) | no indication | Year 12 or below (28%), technical degree (27%), under/post graduate degree (43%) |
| Development of an Electronic Screening and Brief Intervention to AddressPerinatal Substance Use in Home Visiting: Qualitative User-CenteredApproach | Dauber 2022 | no indication | Home visitors: 43% latina, 57% black or african american, 28% other racial or ethnic backgrounds | no indication | no indication | Home visitors: 43% high school education, 29% some college education, 29% graduated from college |
| Usability and acceptability testing of a Plan of Safe Care in a mobilehealth platform | Isaacs 2023 | mean = 30 | White 70%, black or african american 30%, (non hispanic/latino 95%) | no indication | no indication | grade 9-11 20%, high school graduate 35%, junior college degree 5%, some college 35%, some post college work 5% |
| mHealth for maternal mental health: Everyday wisdom in ethical design | Barry 2017 | mean = 40 | no indication | no indication | no indication | no indication |
| Applying Intervention Mapping to develop a community-based interventionaimed at improved psychological and social well-being of unmarried teenagemothers in Uganda | Leerlooijer 2014 | no indication | no indication | no indication | no indication | no indication |
| Participatory design of eHealth solutions for women from vulnerablepopulations with perinatal depression | Gordon 2016 | no indication | no indication | no indication | no indication | no indication |
| Social interaction needs and entertainment approaches to pregnancywell-being in mhealth technology design for low-income transmigrant women:Qualitative codesign study | Aljaberi 2018 | no indication | Caribbean transmigrant | immigrant | minimum wage job | no indication |
| The effect of educational intervention on prevention of postpartumdepression: An application of health locus of control | Moshki 2014 | mean = 28 | no indication | no indication | no indication | illiterate (21%), primary (13%), secondary (32%), higher (33%) |
| Free Time For Wellness: a co-designed intervention utilizing socialnetworks to encourage physical activity for cancer prevention among lowresourced mothers | Houghton 2021 | no indication | 8 LatinX, 1 black, 3 asian, 3 white, 5 not indicated | no indication | no indication | no indication |
| Digital support for living with and beyond gynaecological cancer | Ashmore 2020 | no indication | no indication | no indication | no indication | no indication |
| The Support for New Mums Project: A protocol for a pilot randomizedcontrolled trial designed to test a postnatal psychoeducation smartphoneapplication | Fealy 2019 | no indication | no indication | no indication | no indication | no indication |
| Development of a mobile app to support self-management of anxiety anddepression in African American Women: Usability study | McCall 2021 | mean = 29,8 | Black or African American, and Multiracial (ie, Black or African American and another race) | no indication | no indication | 2 (13%) of them have less than a bachelor's degree, and 13 (87%) have a bachelor's degree or higher |
| Bravo: A physiological indication system for female college students tomanage depression | Liu 2021 | NA | no indication | no indication | no indication | All College-level |
| Conversational Physical Activity Coaches for Spanish and English SpeakingWomen: A User Design Study | Figueroa 2021 | 39,5 | 11% black, 11% white, 77% latina | 27% us, 73% born elsewhere (latin america) | not specified | 5% none, 11% 1-5th grade, 11% 6-8th grade, 11% some high school, 16% all high school, 5% college or technical school, 27% college graduates, 11% graduates |
| A Counseling Mobile App to Reduce the Psychosocial Impact of HumanPapillomavirus Testing: Formative Research Using a User-Centered DesignApproach in a Low-Middle-Income Setting in Argentina | Antelo 2022 | mean = 47.4 years | no indication | no indication | 14 (74%) Economically active (labor force), 5 (26%) Economically inactive (out of labor force) | 12 (63%) Secondary (incomplete and complete), 5 (26%) tertiary incomplete, 2 (11%) Tertiary complete |
| ISDT Case Study of Weâ€™ll App for Postpartum Depression Women | Chiou 2021 | no indication | no indication | no indication | no indication | no indication |
| Designing a tablet-based prematurity education app for parentshospitalized for preterm birth | Rau 2020 | no indication | no indication | no indication | no indication | Varied (in usability tests): high school to graduate degrees. |
| User-Engaged Design in the Adaptation of VIDKIDS Home Visiting Program forVirtual Delivery in the Context of COVID-19 | Duffett-Leger 2020 | no indication | no indication | no indication | no indication | no indication |
| Empowerment of Women Self Help Groups: Human Centered Design of aParticipatory IoT solution | Sreeraj 2020 | no indication | no indication | no indication | no indication | no indication |
| Digital health needs of women with postpartum depression: Focus groupstudy | Lackie 2021 | mean = 34,5 | Chinese 2 (6%) Hispanic or Latin American 1 (3%) South Asian (East Indian, Pakistani, Sri Lankan, etc) 1 (3%) White European 25 (81%) Biracial 2 (6%) | non immigrants | Less than Can $20,000 (US $15,634) 2 (6%) Between Can $20,000 and $39,999 (US $15,634 and $31,266) 2 (6%) Between Can $40,000 and $59,999 (US $31,267 and $46,900) 7 (23%) Between Can $60,000 and $79,999 (US $46,901 and $62,534) 3 (10%) Between Can $80,000 and $99,999 (US $62,353 and $78,167) 4 (13%) Between Can $100,000 and $119,999 (US $78,168 and $92,801) 2 (6%) Between Can $120,000 and $139,999 (US $92,802 and $109,434) 2 (6%) Between Can $140,000 and $159,999 (US $109,435 and $125,068) 2 (6%) More than Can $160,000 (US $125,069) 5 (16%) Prefer not to answer 2 (6%) | Graduated high school or earned GED 2 (6%) Attended some college or university 4 (13%) Graduated 2-year college or university 8 (26%) Graduated 4-year college or university 9 (29%) Postgraduate degree 8 (26%) |
| Effectiveness of smartphone-based mindfulness training on maternalperinatal depression: Randomized controlled trial | Sun 2021 | mean = 29,91 | Han 99.4%, Hui 0.6% | no indication | Family monthly income per person (US$) <309 = 0.7%, 309-618 = 21.1%, 618-926 = 30.3%, â‰¥926 = 48.0 | 15.42 education years |
| Co-designing a lifestyle-focused text message intervention for women afterbreast cancer treatment: Mixed methods study | Singleton 2021 | no indication | no indication | no indication | no indication | no indication |
| Evaluation of an mHealth Intervention (Growinâ€™ Up Healthy Jarjums)Designed With and for Aboriginal and Torres Strait Islander Mothers:Engagement and Acceptability Study | Perkes 2023 | mean = 31.54 | 83% Aboriginal, 15% Nonidentified, 2% Unknown | no indication | no indication | 17% did not finish high school, 32% high school, 27 % certificate, 10% bachelor degree, 7% doploma, 5% postgraduate degree, 2% not applicable |
| Applying User-Centered Design in the Development of a Supportive mHealthApp for Women in Substance Use Recovery | Eaves 2023 | no indication | 50% white, 35% Hispanic, 13% Black, 11% Native American | no indication | History of poverty or housing insecurity | no indication |
| A Web-Based Intervention to Address Risk Factors for Maternal Morbidityand Mortality (MAMA LOVE): Development and Evaluation Study | Amore 2023 | no indication | no indication | no indication | no indication | no indication |
| Yourtime: The development and pilot of a perinatal mental wellbeingdigital tool using a co-design approach | McKellar 2023 | mean = 33 | 3 caucasian, 1 Torres Strait Islander | no indication | no indication | For testing: 14% vocation, 50% university, 35% postgraduate |
| Integrated Design Method and Usability Outcomes of a Mobile Applicationfor Psychological Well-Being During Pregnancy | Perego 2023 | age range between 26-35 | no indication | no indication | no indication | no indication |
| Adaptive Text Messaging for Postpartum Risky Drinking: Conceptual Modeland Protocol for an Ecological Momentary Assessment Study | Dauber 2022 | age range between 18-45 | no indication | no indication | no indication | no indication |
| Promoting Health Behavior Change in the Preconception Period: CombinedApproach to Intervention Planning | Scott 2022 | no indication | no indication | no indication | no indication | no indication |
| Indigenous Women and Their Nutrition During Pregnancy (the Mums and BubsDeadly Diets Project): Protocol for a Co-designed mHealth ResourceDevelopment Study | Gilbert 2023 | no indication | no indication | no indication | no indication | no indication |
| Feasibility and Acceptability of a Mobile Technology Intervention to Support Postabortion Care After Surgical Abortion (the FACTS Study Phase3): Mixed Methods Prospective Pilot Study (Gill 2019 phase 1, Gill 2019 phase 2, Gill 2024 phase 3) | Gill 2024 | Phase 1: mean = 26; Phase 3: mean = 30 | no indication | no indication | Phase I: 69% annual income < CAD $35,000 | Phase I: 36% high school education 38% between high school and bachelor's degree |
| Mobile Mental Health in Womenâ€™s Community-Based Organizations: Protocolfor a Pilot Randomized Controlled Trial | Bhat 2023 (Pilot RCT demographics) | Intervention group: mean =41.6; control group: mean=40.7 | no indication | no indication | Monthly family income: Intervention group: ≤INR 10,000 (US $112.65) 44.9%, > INR 10,000 (US $112.65) 55.1%; Control group: ≤INR 10,000 (US $112.65) 27.8%, > INR 10,000 (US $112.65) 72.2% | Intervention group: No schooling (46.9%), Primary–Middle school (22.5%), Secondary-Higher Secondary (24.5%), Graduation/Diplom (6.1%); Control group: No schooling (52.8%), Primary–Middle school (5.5%), secondary-higher education (30.6%), Graduation/Diploma (11.1%) |
| Improving Mental Health Among Working-Class Indian Women: Insight From AnInterview Study | Reen 2022 | age range between 18 and 54 years old, distributed as follows: 18-24 (3.4%), 25-34 (74.1%), 35-44 (19.3%), and 45-54 (3.2 | no indication | no indication | no indication | 81% of the participants hold a Master's degree, and 19% hold a bachelor's degre |
| A Digital Behavioral Activation Intervention (JuNEX) for Pregnant Women With Subclinical Depression Symptoms: Explorative Co-Design Study | Mancinelli 2024 | guided group (mean = 32.17, n=6); Unguided group (mean = 31, n=5) | no indication | no indication | no indication | Guided group (17% under high-school diploma, 17% high school diploma, 17% bachelor degree, 33% master degree); Unguided group (40% high-school diploma, 40% master degree, 20% specialization (e.g., PhD)) |
| Designing of user interface and user experience in prevent sexual violence application (cekal) using the design thinking method (case study: Region of Bandar Lampung) | Adrian 2024 | no indication | no indication | no indication | no indication | no indication |
| Developing a Guided Web App for Postpartum Depression Symptoms: User-Centered Design Approach | Franco 2024 | mean=32.5 | no indication | no indication | no indication | university education (81.5%), vocational education (10.8%), high school (7.7%) |
| A Chatbot-Based Version of the World Health Organization “Validated Self-Help Plus Intervention for Stress Management: Co-Design and Usability Testing | Fietta 2024 | no indication | no indication | no indication | no indication | no indication |
| User-Centered Design of a Seasonal Calendar App for Women: Insights from a Case Study in India | Menon 2024 | age range between 20-50 | Indian | no indication | no indication | no indication |
| Preconceptional health behavior change in women with overweight and obesity: prototype for SMART strong healthy women intervention. | Materia 2018 | mean = 28.2 | 93% Caucasian, 5% Hispanic, 3% African American | no indication | $40,000–$100,000 (45%), $20,000–$40,000 (18%), <$10,000 (15%), >$100,000 (13%), $10,000–$20,000 (8%) | 4-year degree/grad school (46%), Some college (18%), High school/GED (15%), Other (21%) |
| A Mindfulness-Based App Intervention for Pregnant Women: Qualitative Evaluation of a Prototype Using Multiple Case Studies. (Longitudinal study of 2 papers, another one in the last round) | Rizzi 2025 | no indication | no indication | no indication | no indication, said in the protocal that will be collected | no indication, said in the protocal that will be collected |
| Chatbot to Support the Mental Health Needs of Pregnant and Postpartum Women (Moment for Parents): Design and Pilot Study. | McAlister 2025 | no indication | no indication | no indication | no indication | no indication |
| Align: Empowering High School Students with PMS Awareness and Holistic Support | Dharani 2025 | no indication | no indication | no indication | no indication | no indication |
| Women’s Wellness Guide Using Machine Learning | Mumbaikar 2025 | no indication | no indication | no indication | no indication | no indication |
| Adapting an Anxiety Sensitivity Intervention for Perinatal Mental Health: Development of a Digital Intervention. | Zimmermann 2025 | no indication | Hispanic/Latinx 7 (46.7%), Non-Hispanic/Latinx 6 (40.0%), Don't know 2 (13.3%). White 7 (46.7%), American Indian/Native American, Asian, Black/African American, Native Hawaiian/Pacific Islander 4 (26.6%), More than one race 3 (20.0%), Prefer not to answer 1 (6.7%) | no indication | $0–32,000: 6 (40.0%), $32,001–73,000: 5 (33.3%), ≥$73,001: 3 (20.0%), Prefer not to answer 1 (6.7%) | Some college (<1 year) 4 (26.7%) ≥1 year college, no degree 4 (26.7%), Associate/Bachelor degree 5 (33.3%), Prefer not to answer 2 (13.3%) |
| Mind Ease: An Empathetic AI Companion for Women's Mental Wellness | Subashini 2025 | age range between 18-45 | no indication | no indication | no indication | no indication |
| GETO Application Development: A CulturallyGrounded Gamified Exposure Therapy Platform for Managing Anxiety in Functional Gastrointestinal Disorder | Ardi 2025 | reproductive-age females | no indication | no indication | no indication | no indication |
| Towards effective digital lifestyle interventions for pregnant women with obesity: A qualitative study exploring women's and healthcare providers' perspectives. | de Bruin 2026 | Age range: 27-38 | 9 Dutch, 1 Cape Verdean, 1 Dutch-Indian, 1 Turkish, 1 Moroccan | no indication | no indication, though Socioeconomic diversity was an explicit recruitment goal, but only partially reflected through employment status | no indication |
| Digital Acceptance and Commitment Therapy for Lifestyle Change in Overweight Pregnant Women: A Feasibility Pilot Study | Nicoletti 2026 | no indication | no indication | no indication | no indication | no indication |
| Paripakvata: A Context-Aware Internet of Everything and Artificial Intelligence Integrated Solution for Holistic Pregnancy Care | Pereira 2026 | no indication | no indication | no indication | no indication | no indication |
| A Culturally Sensitive Interactive Digital Narrative to Promote Bodily Awareness Among Afghan Women | Anwar 2025 | Age range: 18-24 | all Afghan women | 2 women live in Afghanistan, 2 live in Sweden, and 1 lives in 'somewhere in EU' | no indication | no indication |
| Translating Combined Mother-Baby Interaction Therapy and Interpersonal Psychotherapy for Postpartum Depression from In-Person to Digital Delivery via MommaConnect. **NOTE: This is a longitudinal study**; Applying the PRECEDE-PROCEED model to develop MommaConnect: a digital healthcare platform for addressing postpartum depression and improving infant well-being | Horowitz 2026 | Age range: 18-44 | 6 women from from general perinetal population, another 6 African American and Black (AA/B) women | no indication | no indication | no indication |
| Identifying the content, capabilities, and design features of a mobile-based cognitive behavioral therapy intervention for managing menopausal symptoms. | Pourshahrokhi 2026 | Age group distribution: 46-50 (42.85%); 51-55 (57.14%); | no indication | no indication | no indication | Bachelor: 3(42.85); Master: 4(57.14) |
| Development of the Healthy Women Intervention to Increase Women's Engagement in Medication Treatment for Opioid Use Disorder: Mixed Methods, User-Centered Design Approach. | Sugarman 2026 | Mean age 38.5 | White 15 (75%), Black or African American 1 (5%), American Indian or Alaskan Native 1 (5%), Other 1 (5%), Prefer not to answer 2 (10%); Hispanic or Latina 5 (25%) | no indication | no indication | Less than high school 3 (15%), High school graduate or GED 4 (20%), Some college 7 (35%), College graduate 6 (30%) |
| Design Process of an Alternate Reality Game (ARG) as a Strategy to Foster Social Support and Well-being of Mothers of Children with ASD | Rodríguez, 2019 | age range between 27-51 | no indication | no indication | no indication | no indication |
| A social media group cognitive behavioral therapy intervention to prevent depression in perinatal youth: Stakeholder interviews and intervention design | Gewali, 2021 | group 1 median age = 22; group 2 median age = 21 | 2 (33.3) African American; 1 (16.7) American Indian or Alaska Native; 1 (16.7) Asian; 3 (50) Latinx; 0 (0) Native Hawaiian or Pacific Islander; 1 (16.7) White; 2 (33.3) Other | no indication | no indication | no indication |
| Effects of the “AI-TA” Mobile App With Intelligent Design on Psychological and Related Symptoms of Young Survivors of Breast Cancer: Randomized Controlled Trial | Jiang, 2024 | control group mean = 40.02; intervention group mean = 40.40 | Chinese women | no indication | Monthly income (CNY ¥), n (%)  Control group (n=55) ITTa group (n=60) respectively <5000 (US $689.72) 11 (20) 11 (18.3) 5000-10,000 (US $689.72-1379.44) 15 (27.3) 17 (28.3) >10,000 (US $1379.44) 29 (52.7) 32 (53.4) | Education level, n (%) Less than or equal to junior college  24 (43.6)  33 (55)  College  26 (47.3)  23 (38.3)    Postgraduate 5 (9.1) 4 (6.7) |
| Co-Designing the MOSAIC mHealth App With Breast Cancer Survivors: User-Centered Design Approach. | Nuseibeh,2024 | 54 | Race and ethnicity, n (%)  White 4 (80)  Black or African American 1 (20) | no indication | no indication | no indication |
| Developing a Mood and Menstrual Tracking App for People With Premenstrual Dysphoric Disorder: User-Centered Design Study. | Apsey, 2024 | age group distribution: 1 participant (5.5%) was aged between 18 and 25 years, 6 were aged between 26 and 33 years, 6 between 34 and 41 years, 4 between 42 and 50 years, and 1 was 50+ | no indication | no indication | no indication | no indication |
| Linguistic Diversity and Mental Well-Being: Co-Designing Custom AI Chatbots with Multilingual Mothers | Elwahsh, 2025 | no indication | no indication | no indication | no indication | no indication |
| Little Cloud: A Digital Companion App to Support Those Experiencing Pregnancy Loss | Wang, 2025 | no indication | Taiwanese | no indication | no indication | no indication |
| A Conceptual Design Framework of GorbhoShongi App for Mental Well-Being Among Bangladeshi Pregnant and Postpartum Women | Ahmed, 2024 | no indication | no indication | no indication | no indication | no indication |
| Tailoring a Skills-Based Serostatus Disclosure Intervention for Transgender Women in South Africa: Protocol for a Usability and Feasibility Study | Daniels, 2025 | no indication | no indication | no indication | no indication | no indication |
| Prevention of Postpartum Depression via a Digital ACT-Based Intervention: Evaluation of a Prototype Using Multiple Case Studies | Nicoletti, 2025 | 34.5 | no indication | no indication | no indication | mean number of years of schooling: 17.88 |
| Design of a mobile application for universal screening for women of child-bearing age engaged in comprehensive addiction and recovery environments (WE-CARE) for substance use and women from the general population. | Shifflett, 2025 | Age group distribution: 18-25 149 26-35 266 36-44 51 | 64% white 36% hispanic or latino | no indication | no indication | no indication |
| Co-designing a mobile application to reduce self-stigma for people with opioid use disorder during pregnancy and the postpartum period. | Osweiler, 2025 | Age group distribution: 18-30 35%; 31-50 13% | white 85% black or african american 15% american indian or alaska native 10% other 5% | no indication | no indication | High school diploma/GED or less 60% Some college, no degree 30% Bachelor’s degree 5% Master’s degree 5% |
| Identifying key indicators to develop a novel mobile application for early screening of postpartum depression in developing countries. | Mustafina, 2025 | Ages ranged 23–38, broken into three bands: 16 participants aged 23–27, 14 aged 28–32, and 15 aged 33–38. | no indication | no indication | no indication | no indication |
